# Rapid target gene validation in complex cancer mouse models using re-derived embryonic stem cells

**DOI:** 10.1002/emmm.201303297

**Published:** 2014-01-15

**Authors:** Ivo J Huijbers, Rahmen Bin Ali, Colin Pritchard, Miranda Cozijnsen, Min-Chul Kwon, Natalie Proost, Ji-Ying Song, Hilda Vries, Jitendra Badhai, Kate Sutherland, Paul Krimpenfort, Ewa M Michalak, Jos Jonkers, Anton Berns

**Affiliations:** 1 Division of Molecular Genetics, The Netherlands Cancer InstituteAmsterdam, The Netherlands; 2 Department of Experimental Animal Pathology, The Netherlands Cancer InstituteAmsterdam, The Netherlands; 3 Division of Molecular Pathology and Cancer Genomics Centre Netherlands, The Netherlands Cancer InstituteAmsterdam, The Netherlands

**Keywords:** cancer, chimeras, embryonic stem cells, mouse models, MycL1

## Abstract

Human cancers modeled in Genetically Engineered Mouse Models (GEMMs) can provide important mechanistic insights into the molecular basis of tumor development and enable testing of new intervention strategies. The inherent complexity of these models, with often multiple modified tumor suppressor genes and oncogenes, has hampered their use as preclinical models for validating cancer genes and drug targets. In our newly developed approach for the fast generation of tumor cohorts we have overcome this obstacle, as exemplified for three GEMMs; two lung cancer models and one mesothelioma model. Three elements are central for this system; (i) The efficient derivation of authentic Embryonic Stem Cells (ESCs) from established GEMMs, (ii) the routine introduction of transgenes of choice in these GEMM-ESCs by Flp recombinase-mediated integration and (iii) the direct use of the chimeric animals in tumor cohorts. By applying stringent quality controls, the GEMM-ESC approach proofs to be a reliable and effective method to speed up cancer gene assessment and target validation. As proof-of-principle, we demonstrate that *MycL1* is a key driver gene in Small Cell Lung Cancer.

## Introduction

The toolbox for generating genetically engineered mouse models (GEMMs) has been steadily growing over the last couple of years. Most of the new technologies deal with genetic modification methods of embryonic stem cells (ESCs), for instance using a recombinase or an integrase to introduce genetic elements in predefined loci (Belteki *et al*, 2003; Beard *et al*, 2006; Seibler *et al*, 2007) or Zinc finger, TAL effector and RNA-guided nucleases to create mutant alleles with high flexibility and ease (Urnov *et al*, 2010; Miller *et al*, 2011; Cong *et al*, 2013). Although these technologies increase versatility, they provide minor time gains as the time spent to genetically engineer ESCs is often dwarfed by the time required to cross the resulting mice to existing GEMMs in order to obtain the final experimental cohort. This is particularly an issue in cancer research, as spontaneous tumor development in GEMMs often requires the (in)activation of multiple genetically modified oncogenes and tumor suppressor alleles. For instance, we have developed GEMMs for Small Cell Lung Cancer (SCLC) and mesothelioma that have four and six conditional alleles, respectively (Meuwissen *et al*, 2003; Jongsma *et al*, 2008). This genetic complexity has hampered the use of GEMMs to study additional candidate cancer genes for their role in tumor initiation and progression. This problem will only become more pressing now genome-wide association studies and genetic screens result in the identification of an increasing number of candidate genes whose roles in tumorigenesis need confirmation in relevant *in vivo* models (Chin *et al*, 2011).

One strategy to accelerate target gene validation in mouse models is to apply the CRISPR/Cas system in zygotes for the one-step generation of animals carrying mutations in multiple genes (Wang *et al*, 2013). Though extremely powerful, this technique needs to be further developed to allow for the controlled introduction of transgenes and/or conditional alleles. Also, off-target effects are likely to occur and need to be taken into account (Fu *et al*, 2013). We, and others have presented an alternative and well-controlled strategy by re-deriving ESCs from well-established and validated GEMMs and use these GEMM-ESCs as the basis for further genetic engineering either by classic gene targeting, gene editing or recombinase-mediated transgene integration (Nichols *et al*, 2009; Huijbers *et al*, 2011; Premsrirut *et al*, 2011). These GEMM-ESCs contain the same genetic modifications as present in the original model plus the newly introduced genetic modification, for instance a frequently observed point mutation in a tumor suppressor gene or a transgene with conditional expression of an oncogene that is often amplified or otherwise overexpressed in a particular tumor type. These modified GEMM-ESCs can be used to generate high quality chimeras that are likely equally susceptible to tumor induction as the original GEMM and serve as a defined experimental cohort differing only by the introduced modification. Main advantages of this approach are speed and flexibility, as it permits comparative analysis of phenotypic consequences of different genes and allelic series in a particular GEMM. Instead of crossing the chimeric mice to the desired strain and genetic background, ready-to-use GEMMs can now be produced on-demand. This reduces both costs and total number of mice needed per experiment, as establishing and maintaining a large breeding colony is expensive and always leads to surplus animals that cannot be used in experiments. Furthermore, genetic drift is prevented as the same GEMM-ESCs lie at the basis of each experimental cohort for a particular cancer type. This approach also allows for the establishment of a GEMM-ESC bank for distribution ESCs with complex genotypes; a resource that likely gives a new impulse to the generation of custom-made mouse models either for preclinical use or cancer gene validation.

The feasibility of the GEMM-ESC production approach depends on reliable procedures and robust quality controls, including (i) ESC culture procedures that guarantee maintenance of pluripotency; (ii) monitoring of the genomic stability of ESCs; (iii) procedures for routine production of chimeras with a major contribution of the GEMM-ESCs to different tissues. The chimeric lines should also be germline-competent to facilitate the production of permanent lines if desirable. Here, we present the performance of the GEMM-ESC approach based on three different GEMMs, two models for lung cancer and one for mesothelioma. We employed the Flp-mediated integration technology (Flp-in) as proof-of-concept for genetic engineering of GEMM-ESCs. Finally, we apply the GEMM-ESC approach to validate *Mycl1* as a bona fide oncogene in SCLC.

## Results

### ESC culture and pluripotency

The first step in the GEMM-ESC approach is the derivation of ESC from the desired GEMMs, which are often backcrossed to a specific strain background, such as C57BL/6J (black coat color) or FVB/n (white coat color). Using classical culture conditions, ESC derivation can be achieved for permissive strains, such as 129 and C57BL/6N, but various strains are thought to be non-permissive (Kawase *et al*, 1994; Schoonjans *et al*, 2003). New culture protocols now permit the derivation of ESCs from refractory strains (Ying *et al*, 2008). In these protocols, culture media containing fetal bovine serum (FBS) and feeder cells are replaced by defined N2B27 medium supplemented with two inhibitors (2i): the MEK1 inhibitor, PD0325901, which effectively blocks MEK/ERK signaling thereby preventing ESC differentiation, and the GSK3 inhibitor, CHIR99021, which acts as a Wnt agonist thereby stimulating growth of ESCs. We used this 2i medium to derive ESCs from wild-type C57BL/6J and FVB/n strains and obtained 4 and 12 ESC clones, respectively (Table 1). Culturing of ESCs in 2i medium improved their overall quality. Expression analysis of the core ESC transcription factors Nanog, Oct4 (also known as *Pou5f1*) and Sox2 in wild-type 129/Ola ESCs showed a higher percentage of naïve, Nanog^pos^;Oct4^pos^;Sox2^pos^ ESCs under the new culture conditions as compared to the classic conditions (supplementary Fig S1). These results demonstrate that 2i medium is more effective in maintaining ESCs in a naïve, undifferentiated state.

**Table 1:** ESC derivation.

Genotype	Strain	Embryos	ICM	ESC clones	Efficiency^a^(%)	Male	Female
Wt	C57LB/6J	30	17	4	13.3	4	0
Wt	FVB/n	35	22	12	34.3	6	6
*Kras*^*LSL-G12D*^	C57BL/6J	57	24	8	14.0	8	0
*Rb1*^*F/F*^ *;Trp53*^*F/F*^	FVB/n;129/Ola	145	63	13	8.9	13	0
*Nf2*^*F/F*^ *;Trp53*^*F/F*^ ;Cdkn2a^^*^/^*^^	FVB/n;129/Ola	65	31	5	7.7	5	0
*Rb1*^*F/F*^ *;Trp53*^*F/F*^ *;Col1A1-frt*	FVB/n;129/Ola	32	26	5	15.6	3	2

a ESC derivation efficiency: percentage of isolated embryos resulting in established ESC clones.

### Quality controls of derived ESCs

**Figure 1:**
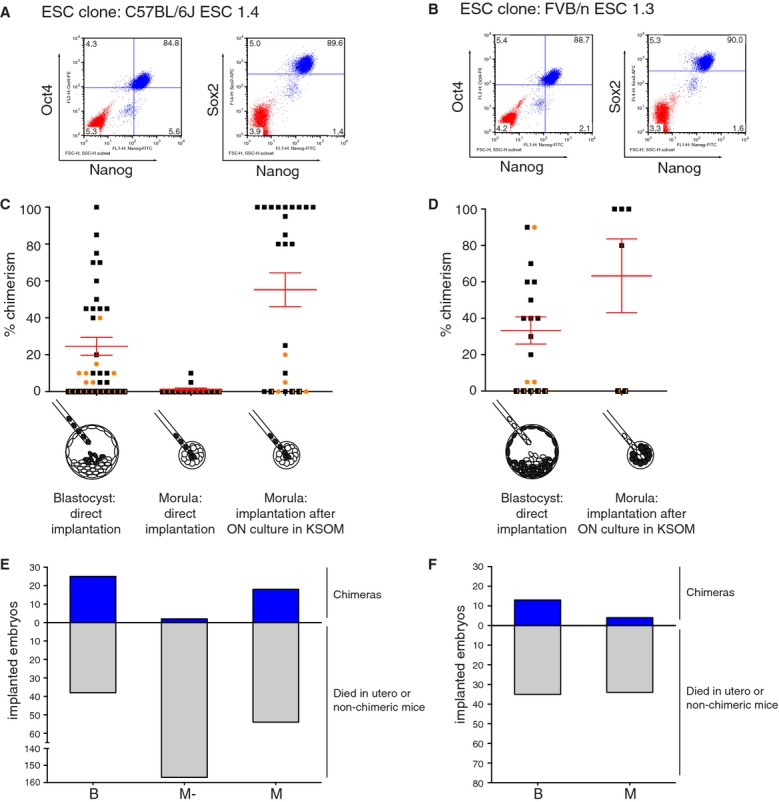
Optimization of ESC culture and injection procedures. A FACS profile of three core ESC transcription factors, Nanog, Oct4 and Sox2, in C57BL/6J ESC clone 1.4cultured in 2i medium (Blue). Red population represents the isotype control. B BFACS profile of three core ESC transcription factors, Nanog, Oct4 and Sox2, in FVB/n ESC clone 1.3 cultured in 2i medium (Blue). Red population represents the isotype control. C Three ESC injection procedures for C57BL/6J ESC clone 1.4 were evaluated on basis of chimeric contribution. Injecting 4–8 ESC per FVB/n morula followed by overnight culture in KSOM medium provided the best chimeras, with nine mice showing 100% coat color contribution (entirely black). 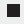 male, 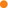 female, 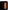 n.d. D Two ESC injection procedures for FVB/n ESC clone 1.3 were evaluated on basis of chimeric contribution. Blastocyst injections resulted in reasonable chimeras whereas ESC injections into morulae in combination with overnight culture improved chimerism with three out of four live borns showing 100% chimerism (entirely white). The 80% chimera was a runt and died before weaning. 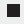 male, 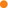 female, 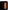 n.d. E,F Efficiency of ESC injection procedures shown in (C) and (D) based on number of viable chimeras born compared to the total number of implanted embryos for C57BL/6J ESC clone 1.4 (E) and FVB/n ESC clone 1.3 (F). Note, for both ESC clones fewer chimeras were observed relative to the total number of implanted embryos when comparing ESC injected morulae to ESC injected blastocysts.

The quality of derived ESCs was assessed on the basis of three criteria: expression of stem cell markers, chimeric contribution and germline transmission. The first criterion was determined by FACS profiling. ESC clones from either C57BL/6J (clone 1.4) or FVB/n (clone 1.3) showed robust expression of Nanog, Oct4 and Sox2 in the majority of cells (Fig 1A and B). To test chimeric contribution of the derived ESCs, each clone was injected into host blastocysts or morulae to generate chimeric animals that were scored for their coat-color chimerism. When ‘black’ C57BL/6J ESCs were injected in ‘white’ FVB/n hosts, the coat color of the resulting chimeras was scored for the absence of white fur and *vice versa*. We compared three injection strategies for the wild-type C57BL/6J ESC clone: (i) blastocysts injected with 12–15 cells with direct implantation into foster mothers; (ii) morulae injected with 4–8 cells with direct implantation; (iii) morulae injected with 4–8 cells with implantation after overnight culture of the embryos in embryo culture medium (Fig 1C). Based on the coat color contribution the latter strategy clearly outperformed the other two, with 100% coat color contribution for several mice. Similar results were obtained for a wild-type FVB/n ESC clone (Fig 1D). The increase in coat color contribution came at a price, as the number of liveborn chimeras relative to the total number of implanted embryos was lower for the morula injections than for the blastocyst injections (Fig 1E and F and supplementary Table S1). This drop in viability is likely the result of aberrant embryonic development leading to resorption *in utero* or in milder cases the birth of runted animals. In addition, some foster mice implanted with morulae injected with FVB/n or FVB/n;129/Ola ESCs were unable to give natural birth and caesarean section was required. To ensure a practical workflow, we decided to inject C57BL/6J ESC clones into morulae (FVB/n) as for this background the benefit of improved chimerism outweighed the complications. In case of FVB/n or mixed FVB/n;129/Ola ESC clones we used more fail-safe blastocyst injections (C57BL/6N).

The most stringent quality control for ESC lines is the ability of the chimeras to give germline transmission (GLT). Although strictly speaking not required for an approach in which chimeras serve as an endpoint, we decided to maintain this quality control as a means to identify ESC clones with impaired germline-competence caused by loss of pluripotency or the acquisition of genetic defects during culture and manipulation. We observed efficient GLT for chimeras obtained from both the C57BL/6J and the FVB/n ESC clones regardless of the injection procedure (supplementary Table S1).

### Derivation of germline-competent ESCs from mouse models with complex genotypes

Two GEMMs of human lung cancer were selected for the derivation of ESCs: the *Kras*^*LSL-G12D*^ non small cell lung cancer (NSCLC) model and the *Rb1*^*F/F*^ *;Trp53*^*F/F*^ small cell lung cancer (SCLC) model (Jackson *et al*, 2001; Meuwissen *et al*, 2003). These mice develop lung tumors after switching of the conditional alleles by a Cre recombinase introduced in the target cells via adenovirus (Ad5-Cre) intubation in the lung. ESCs were also derived from *Nf2*^*F/F*^ *;Trp53*^*F/F*^ ;Cdkn2a^*/*^ mice, which carry—in addition to conditional *Nf2* and *Trp53* alleles—a homozygous mutation in *Cdkn2a* that results in loss of p16^Ink4a^ expression but retention of the alternative reading frame protein p19^Arf^ (Krimpenfort *et al*, 2001). *Nf2*^*F/F*^ *;Trp53*^*F/F*^ ;Cdkn2a^*/*^ mice develop invasive mesotheliomas after intrathoracic Ad5-Cre injection due to loss of Nf2 and p53 in the mesothelial lining (Jongsma *et al*, 2008). The NSCLC model was maintained on a C57BL/6J background, whereas the SCLC and mesothelioma models were on a mixed FVB/n;129/Ola background. As expected, the efficiency of ESC derivation was similar between genotypes and comparable to the two wild-type strains (Table 1). The gender of the derived ESC clones was determined by Y-chromosome specific PCR. We observed a strong bias towards male ESC clones (Table 1), which was likely caused by reduced morphological appearance and growth of female ESC clones, resulting in their discontinuation early in the derivation process. Only in cases where we decided to expand all clones, e.g. wild-type FVB/n, we obtained both male and female clones. At later passage, these female ESC clones caught up and were indistinguishable from male ESC clones on basis of growth and morphology. However we restricted ourselves to male ESC clones as it has been reported that female lines derived from inbred strains often loose one of the two X chromosomes during expansion (Barakat & Gribnau, 2010).

**Figure 2:**
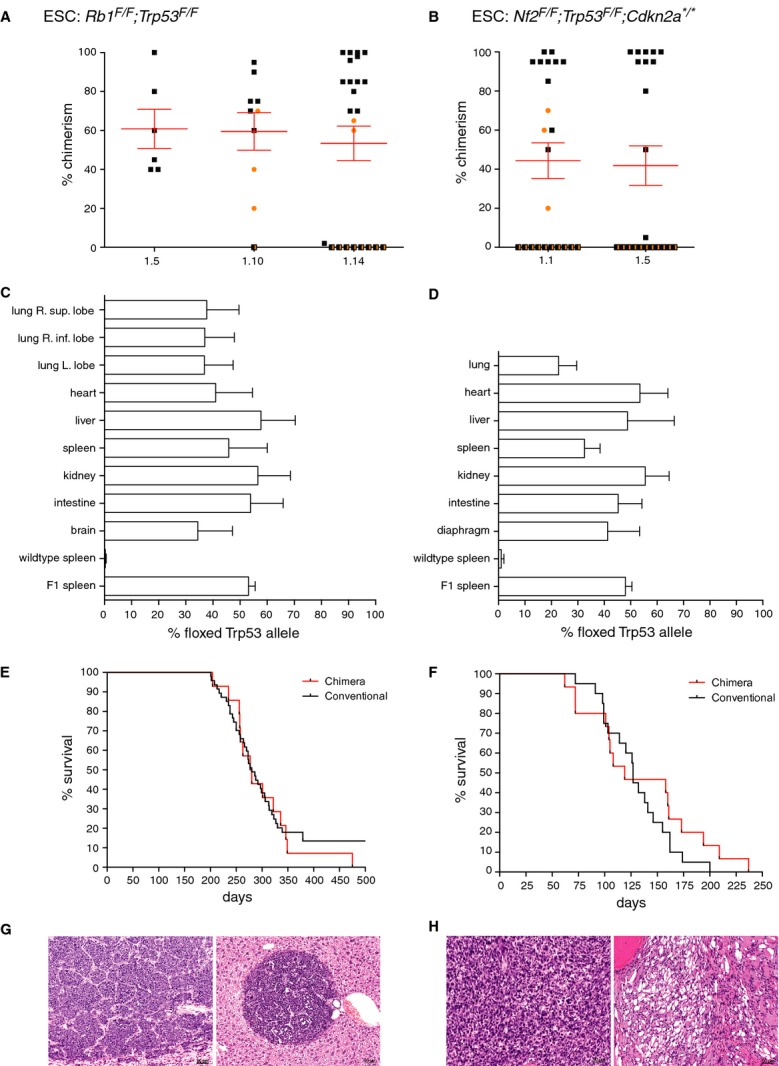
Validation of chimeras. A,B Three *Rb1*^*F/F*^ *;Trp53*^*F/F*^ ESC clones (A) and two *Nf2*^*F/F*^ *;Trp53*^*F/F*^ ;Cdkn2a^*/*^ ESC clones (B) were injected into C57BL/6N blastocysts and scored for their chimeric contribution. All ESC clones gave reasonable numbers of chimeric animals relative to the implanted embryos (supplementary Table S1) and, as expected, most of the chimeras were males as we exclusively used male ESC clones. 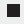 male, 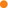 female, 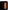 n.d. C,D Comparison between chimeric contribution estimated on basis of coat-color versus genetic chimerism, tested in various tissues. Southern blot analysis was performed with a probe that distinguishes between a wild-type *Trp53* allele or the floxed *Trp53* allele reflecting the contribution by the host ESCs or cultured ESCs, respectively (example in supplementary Fig S3). Controls are wild-type spleen (0% chimerism expected) and F1 offspring of chimeras (50% chimerism expected). (C) Genetic chimerism of *Rb1*^*F/F*^ *;Trp53*^*F/F*^ chimeras with coat color chimerism ranging from 70 to 100% (average 84%, *n* = 7). (D) Genetic chimerism of *Nf2*^*F/F*^ *;Trp53*^*F/F*^ ;Cdkn2a^*/*^ chimeras with coat color chimerism ranging from 85 to 100% (average 95%, *n* = 4). E Survival curves of *Rb1*^*F/F*^ *;Trp53*^*F/F*^ mice intratracheally injected with Ad5-Cre. Black line, conventional mice; red line, chimeras. F Survival curves of *Nf2*^*F/F*^ *;Trp53*^*F/F*^ ;Cdkn2a^*/*^ mice intrathoracically injected with Ad5-Cre. Black line: conventional mice; Red line: chimeras. G Typical example of a neuroendocrine carcinoma (Small Cell Lung Cancer) in the lung (left panel) and a metastatic lesion in the liver (right panel). H Typical example of a mesotheliomatous lesion in the thoracic cavity. Tumor cells are either spindle sarcomatoid cells (left panel) or vacuolated epithelioid cells (right panel).

Three *Rb1*^*F/F*^ *;Trp53*^*F/F*^ ESC clones, two *Nf2*^*F/F*^ *;Trp53*^*F/F*^ ;Cdkn2a^*/*^ clones and one *Kras*^*LSL-G12D*^ clone were tested for their contribution to chimeras. All clones gave reasonable numbers of chimeric animals relative to the implanted embryos and, as expected, most of the chimeras were males (supplementary Table S1). Most chimeras were of high quality, showing coat-color chimerism of more than 70% (Fig 2A and B, supplementary Fig S2A) and efficient GLT in the first litter (supplementary Table S1).

### Contribution of derived ESCs to various organs of chimeric mice is extensive and allows for efficient tumor induction

One of the key features of the GEMM-ESC approach is the ability to directly evaluate tumor phenotypes in chimeric mice, bypassing the need for any breeding. The success of this approach depends on the contribution of cultured ESCs to the various tissues in the chimeric mice. To assess this, we performed Southern blot analysis on genomic DNA extracted from multiple tissues of chimeric mice from two independent GEMMs. To determine the level of genetic chimerism, we used a probe that distinguishes between a wild-type *Trp53* allele and the floxed *Trp53*^*F2-10*^ allele (Jonkers *et al*, 2001), reflecting the contribution by the host ESCs or cultured ESCs, respectively (supplementary Fig S3). The contribution of the cultured ESCs was comparable for most organs with the exceptions of the lung and brain, which consistently scored the lowest (Fig 2C and D). In general the percentage of coat-color chimerism was scored higher than the genetic chimerism.

A potential limitation of the GEMM-ESC approach is that chimeric mice have a smaller target cell population for oncogenic transformation. To determine whether this influences tumor type, latency and incidence a comparison was made between conventional mice and chimeras for the three GEMMs (Fig 2E and F and supplementary Fig S2B). For the SCLC and mesothelioma models the tumor type, incidence and latency was identical between the conventional and the chimeric mice (Fig 2E–H). All chimeric mice from the SCLC model developed lung neuroendocrine carcinomas resembling SCLC, often with invasion to the mediastinum and metastases to the liver (Fig 2G). All chimeric mice from the mesothelioma model developed epithelioid, sarcomatoid or biphasic mesotheliomas that were highly invasive into nearby tissues (Fig 2H). In the NSCLC model, the incidence and tumor types in the chimeric mice was again identical to the incidence and tumor types observed in the original strain. All NSCLC chimeras developed multiple lesions in the lung ranging from adenomatous or bronchioalveolar hyperplasia to bronchioalveolar adenomas, adenocarcinomas and papillary carcinomas (supplementary Fig S2B and C). Surprisingly, the tumor latency was shorter for the NSCLC chimeras than for the conventional mice. It is possible that host-derived FVB/n cells in the lung create a tumor-permissive or pro-tumorigenic microenvironment. Alternatively, as the NSCLC chimeric cohort was produced from a single ESC clone, an unidentified genetic lesion might have been acquired during the re-derivation process that leads to accelerated tumor growth. Combined, these data illustrate that tumor induction in chimeras is as efficient as in animals carrying the conditional lesions in all of their cells. The GEMM-ESC approach is therefore a very effective strategy to swiftly generate cohorts of mice for *in vivo* tumor studies.

### Targeting of GEMM-ESCs under 2i culture conditions is efficient but requires genetic and phenotypic quality control

**Figure 3:**
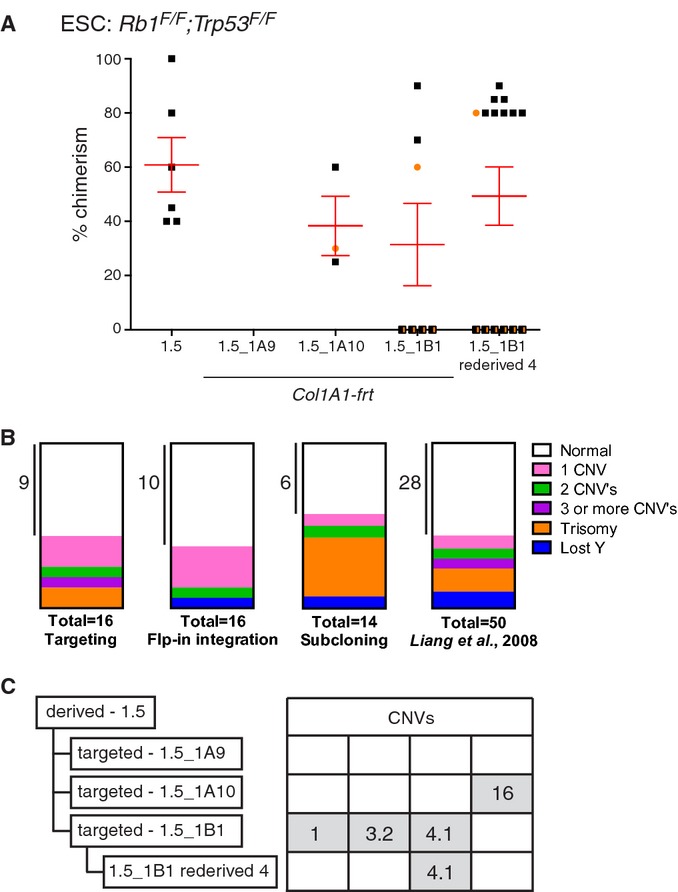
Genomic stability of targeted GEMM-ESC clones. Comparison of chimeric contribution between the parental *Rb1*^*F/F*^ *;Trp53*^*F/F*^ ESC clone 1.5 and three *Col1a1-frt* targeted derivatives. Correct targeting was confirmed by Southern blot analysis using a 3′ probe in the *Col1a1* locus (supplementary Fig S4A and B). Two *Col1a1-frt* targeted clones, i.e. 1.5_1A10 and 1.5_1B1, provided good and germline-competent chimeras (supplementary Table S1). One chimera from the *Rb1*^*F/F*^ *;Trp53*^*F/F*^ ESC clone 1.5_1B1 was backcrossed twice to the original strain and ESC were re-derived, i.e. clone 1.5_1B1 re-derived 4 (Table 1). This ESC clone resulted in improved chimeras compared to the parental clones. 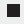 male, 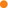 female, 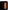 n.d.Parts of whole representation of genetic aberrations observed in GEMM-ESCs cultured in 2i medium and subjected to either gene targeting, Flp-in integration and subcloning (supplementary Table S4). Last box represent the genetic aberrations observed in ESCs cultured under classic culture conditions as reported by Liang *et al*, 2008.Summary of CNVs observed in *Rb1*^*F/F*^ *;Trp53*^*F/F*^ ESC clones as detected by aCGH. Two *Col1a1-frt* targeted clones acquired four independent CNVs. Some CNVs can be transmitted via the germ line as CNV-4.1 was maintained after backcrossing twice to the original strain, see ESC clone 1.5_1B1 re-derived 4. A detailed description of all CNVs is provided in supplementary Table S3.

The second step in the GEMM-ESC approach involves targeting of GEMM-ESCs with a Flp-in module just after the 3′UTR of the *Col1a1* locus (Beard *et al*, 2006). This module, named *Col1a1-frt*, serves as a docking site for introduction of transgene-coding plasmids by Flp recombinase-mediated integration. We choose this system as it is successfully applied by others (Zhu *et al*, 2009; Yilmaz *et al*, 2012), allows for transgene induction in multiple somatic cell types (Carey *et al*, 2010) and is compatible with a vector system for doxycycline-regulated, fluorescence-linked shRNAs (McJunkin *et al*, 2011; Premsrirut *et al*, 2011; Dow *et al*, 2012). Targetings were performed under the new 2i culture conditions; colonies were screened by PCR and correctly targeted clones were confirmed by Southern blotting (supplementary Fig S4A and B). For all three genotypes, i.e. *Rb1*^*F/F*^ *;Trp53*^*F/F*^, *Nf2*^*F/F*^ *;Trp53*^*F/F*^ ;Cdkn2a^*/*^ and *Kras*^*LSL-G12D*^, similar targeting efficiencies of ˜35% were achieved (supplementary Table S2). These efficiencies were comparable to that of the wild-type 129/Ola ESC clone IB10 (36% under 2i culture conditions). The *Col1a1-frt* targeted GEMM-ESC clones were subsequently injected into morulae or blastocysts to produce chimeric mice. Out of 11 clones injected, three failed to produce chimeras. All other clones produced germline-competent chimeras (Fig 3A, supplementary Fig S5A and B, Table S1).

Assessment of the genomic integrity of these targeted clones by array comparative genomic hybridization (aCGH) revealed three types of genomic aberrations, either small copy number variations (CNVs) in the 0.2–1.0 megabase (Mb) range, loss of the Y-chromosome or trisomy of entire chromosomes (supplementary Fig S5C and D, Tables S3 and S4). These types of aberrations have also been reported by others with similar frequencies in targeted wild-type ESC clones cultured under classic culture conditions (Fig 3B; Liang *et al*, 2008). All CNVs showed a single copy gain and were non-recurrent, neither in our tested GEMM-ESC clones nor in the published dataset (Liang *et al*, 2008), indicating that there is no strong biological selection for specific amplifications or deletions. For future experiments we selected targeted GEMM-ESC clones with no or few CNVs of <1 Mb each. Note that there is an option to ‘clean-up’ a targeted GEMM-ESC clone with several CNVs by re-deriving ESC clones from decedents of chimeras backcrossed to the original GEMM. For example, re-derivation of ESC clones from second-generation descendants of the *Col1a1-frt* targeted *Rb1*^*F/F*^ *;Trp53*^*F/F*^ ESC clone 1B1 resulted in loss of two of the three CNVs present in the original clone (Fig 3C). This clone was used to generate chimeras and outperformed the original targeted clone 1B1 based on the number of chimeras and percentage of coat-color chimerism (Fig 3A).

### Efficient introduction of reporter constructs via Flp recombinase mediated integration

The third and final step in the GEMM-ESC approach is the introduction of a transgenic construct in the *Col1a1-frt* locus using the Flp recombinase. As proof-of-principle, we generated two genetic inversion reporter constructs, called *frt-invCAG-Luc* or *frt-invEF1-Luc*, containing a codon-optimized firefly Luciferase 2 ( *Luc*) gene that—following Cre-mediated inversion—is expressed from a constitutive *CAG* or *EF1a* promoter, respectively (supplementary Fig S4C). These vectors were introduced into a *Col1a1-frt* targeted *Nf2*^*F/F*^ *;Trp53*^*F/F*^ ;Cdkn2a^*/*^ GEMM-ESC clone with 100% efficiency. The same efficiency was achieved for Flp-in of the *frt-invCAG-Luc* construct into the *Col1a1-frt;Rb1*^*F/F*^ *;Trp53*^*F/F*^ GEMM-ESC clone (supplementary Table S2, Fig S4D). Five out of seven ESC clones used for injections into pre-implantation embryos performed well and gave chimeric animals that showed in most cases >50% coat-color chimerism (supplementary Fig S5E and F). PCR screening of offspring from crosses of male chimeras with the original GEMM revealed that all tested chimeras gave GLT of the Luciferase allele (supplementary Table S1).

### *In vivo* imaging of tumor growth in chimeric reporter mice

**Figure 4:**
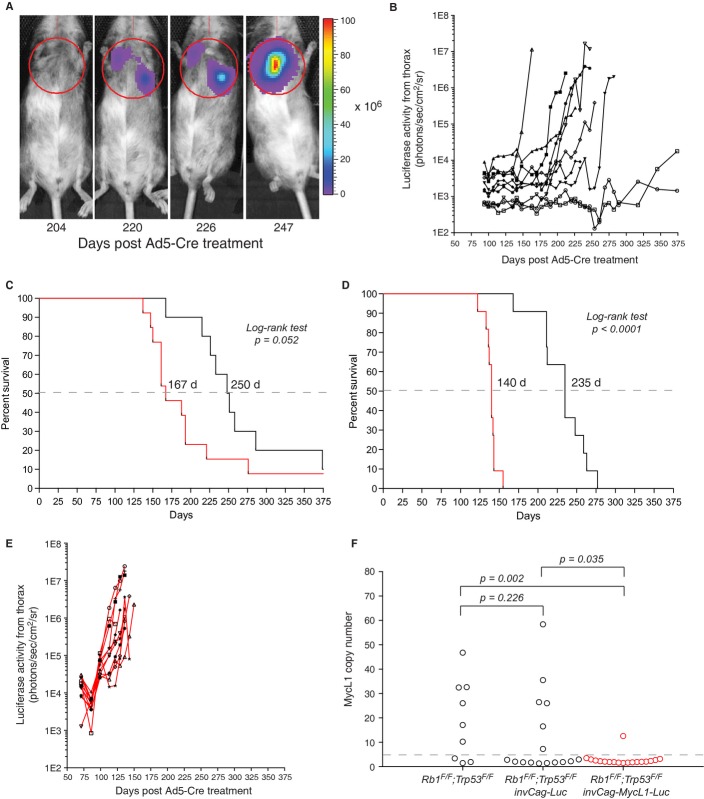
Luciferase imaging of SCLC in chimeras. *In vivo* imaging of a *invCAG-Luc;Rb1*^*F/F*^ *;Trp53*^*F/F*^ chimeric mouse injected intrathoracically with Ad5-Cre. Tumor growth was monitored weekly by bioluminescence imaging.Luciferase activity emitted from the thorax of 10 chimeric *invCAG-Luc;Rb1*^*F/F*^ *;Trp53*^*F/F*^ mice. Each line represents measurements of an individual mouse. The chimeric mouse with the lowest coat-color chimerism (○, 20%) did not develop a tumor, while the second lowest chimera (□, 35%) did develop SCLC though with a long latency. One chimera (♦, 962975) failed to show any Luciferase activity but did develop SCLC. Analysis of the tumor revealed a lack of Cre-mediated switching of the *invCag-Luc* transgene (supplementary Fig S7).Survival curves of chimeric *Rb1*^*F/F*^ *;Trp53*^*F/F*^ mice containing either the *invCag-Luc* (black line) or the *invCag-MycL1-Luc* (red line) transgene, intratracheally injected with Ad5-Cre. Median survival indicated by the dotted line was 250 and 167 days, respectively.Survival curves of F1 *Rb1*^*F/F*^ *;Trp53*^*F/F*^ mice containing either the *invCag-Luc* (black line) or the *invCag-MycL1-Luc* (red line) transgene, intratracheally injected with Ad5-Cre. Median survival indicated by the dotted line was 235 and 140 days, respectively.Luciferase activity emitted from the thorax of 11 F1 *invCAG-MycL1-Luc;Rb1*^*F/F*^ *;Trp53*^*F/F*^ mice. Each line represents measurements of an individual mouse.MycL1 copy number in SCLC tumors from three different genotypes determined by real-time PCR and aCGH. Each circle represents a primary SCLC tumor. All tumors with more than four copies (dotted line) were considered positive for MycL1 amplification. Note that overexpression of *MycL1* by the transgene significantly reduces the frequency of genomic MycL1 amplifications in tumors as compared to the *Rb1*^*F/F*^ *;Trp53*^*F/F*^ control ( *P* = 0.002 Fisher's Exact Test) and the *invCAG-Luc;Rb1*^*F/F*^ *;Trp53*^*F/F*^ control ( *P* = 0.035 Fischer's Exact Test).

The chimeric animals with the new Luciferase reporter constructs were treated with Ad5-Cre to induce tumor formation. The majority of the *invCAG-Luc;Rb1*^*F/F*^ *;Trp53*^*F/F*^ chimeras developed SCLC with similar latency as presented earlier (Figs 4A–C and 2E). One mouse with the lowest coat-color chimerism failed to develop a tumor after 375 days, possibly reflecting insufficient contribution of GEMM-ESCs to lung epithelium for reliable use in experimental cohorts (Fig 4B). Bioluminescence imaging of luciferase activity revealed tumor initiation at variable time points, ranging between 140 and 320 days, after which the majority of tumors displayed exponential growth until animals had to be sacrificed because of respiratory distress. In the mesothelioma model the results were less pronounced. Here, all but one chimera developed mesothelioma with thoracic Luciferase expression; however, the increase in Luciferase expression over time was limited and in some cases leveled off after an initial increase (supplementary Fig S6). This occurred for both reporter constructs, but was most often observed for the reporter construct carrying the *EF1a* promoter. The underlying cause for this behavior remains speculative and could have multiple reasons. It might be due to quenching of the luminescence signal by pleural effusion, i.e. accumulated liquid in the pleural cavity. Also, the immunogenicity of the Luciferase protein might trigger an immune response against Luciferase-expressing tumor cells, leading to selective outgrowth of tumor cells with low or no luciferase expression (Jeon *et al*, 2007). Thirdly, the *CAG* and *EF1a* promoters might be silenced by methylation. We have indications that at least the latter event occurs, as treatment of cultured primary mesothelioma cells derived from a chimeric animal with the demethylating agent, 5-aza-2dC, resulted in a marked increase in Luciferase expression (supplementary Fig S6E). Promoter silencing is likely due to the presence of bacterial DNA of the plasmid integrated in the *Col1A1* locus (Tasic *et al*, 2011). Still, the promoter silencing appears to be model or cell type dependent, as tumors in the SCLC model showed robust Luciferase expression judged by the exponential increase in luminescence signals measured in the majority of the *invCAG-Luc;Rb1*^*F/F*^ *;Trp53*^*F/F*^ chimeras (Fig 4B). In the few cases where no Luciferase expression was observed in SCLC tumors, the *invCag-Luc* transgene had failed to recombine after Cre expression (supplementary Fig S7).

### *In vivo* validation of Mycl1 as a bona fide oncogene in SCLC

Tumors of small cell lung cancer patients often show amplifications of genomic regions coding for either *MYCL1*, *c-MYC* or *NMYC* (Iwakawa *et al*, 2013). In SCLC tumors of the *Rb1*^*F/F*^ *;Trp53*^*F/F*^ model, amplifications of the genomic region 4qD2.2 coding for *Mycl1* are frequently observed (Calbo *et al*, 2005; Dooley *et al*, 2011). To confirm that the *Mycl1* oncogene plays a causal role in the progression of SCLC we adapted our *frt-invCAG-Luc* construct by introducing the *Mycl1* cDNA and an internal ribosomal entry site (IRES) upstream of the *Luc* gene (supplementary Fig S8A). This construct, named *frt-invCAG-Mycl1-Luc*, allows for simultaneous expression of both Mycl1 and Luc after Cre recombination and was introduced in the re-derived *Col1a1-frt* targeted *Rb1*^*F/F*^ *;Trp53*^*F/F*^ ESC clone 1B1 (Fig 3) with high efficiency (supplementary Table S2). Two ESC clones were used to generate chimeras (supplementary Table S1 and Fig S8B). These chimeras were treated intratracheally with Ad5-Cre and developed neuroendocrine carcinomas in lung with a considerably shorter latency as compared to the *invCAG-Luc;Rb1*^*F/F*^ *;Trp53*^*F/F*^ chimeras, with a median survival of 167 days as opposed to 250 days (Fig 4C). This tumor acceleration was even more pronounced in the F1 cohorts, which showed a median survival of 235 days for *invCAG-Luc* and 140 days for *invCAG-Mycl1-Luc* (Fig 4D), highlighting the importance of *Mycl1* in SCLC development. This additional decrease in tumor latency is likely caused by the increase in target cell population in the F1 mice as compared to the chimeras, especially since lung tissue showed the least contribution of GEMM-ESCs in chimeric mice (Fig 2C). This phenomenon, which we did not observe in our previous cohorts, might relate to the average quality of chimeras, as for this experiment we induced tumors in chimeric mice with a wide range (5–95%) of coat-color chimerism (supplementary Fig S8B). Monitoring of Luciferase expression in individual mice from the F1 cohort revealed that all Mycl1 expressing tumors initiated around the same time point and showed identical growth leftacteristics, making this model very suitable for tumor intervention studies (Fig 4E). To proof that *Mycl1* is the driver gene for the 4qD2.2 amplicon, we verified *Mycl1* amplification status in genomic DNA of tumors from control *Rb1*^*F/F*^ *;Trp53*^*F/F*^ mice, or from mice with either the *invCAG-Luc* or the *invCAG-Mycl1-Luc2* transgene (both chimeras and F1) by low-coverage DNA sequencing and real-time PCR (Fig 4F). *Mycl1* was amplified in 37.5–66.7% of the control tumors, whereas it was only amplified in 6% of the *Mycl1* transgenic tumors, clearly validating *Mycl1* as a driver for SCLC development.

## Discussion

**Figure 5:**
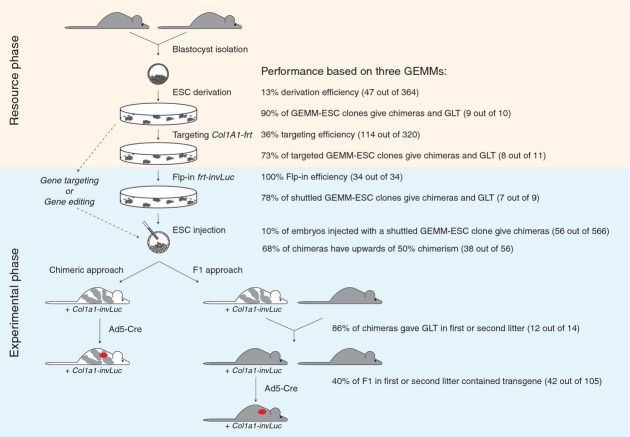
Efficiency of the GEMM-ESC approach.Schematic representation of the GEMM-ESC approach including the performance of the individual steps. The approach is divided in two phases: a resource phase and an experimental phase. The resource phase includes ESC's derivation and targeting with the *Col1a1-frt* vector, performed once per GEMM and takes ˜6 months, including the necessary quality controls. The experimental phase is mainly focused on introducing a transgene-coding plasmid in a validated GEMM-ESC clone using the Flp-in method that allows for consecutive manipulations and takes ˜4 months to obtain a chimeric cohort. Alternatively, GEMM-ESC clones are also suitable for direct targeting of a specific gene or the introduction of mutant alleles using gene editing (arrows with dotted lines). The experimental phase also includes the option to follow an F1 route as almost all GEMM-ESC clones showed germline transmission (GLT). In practice, we advise that for each model (i) multiple *Col1a1-frt* targeted GEMM-ESC clones are screened for their ability to efficiently generate high quality chimeras, (ii) two of the best-performing clones are selected for the Flp-in procedure, and (iii) at least two transgene-coding GEMM-ESC clones are used to generate cohorts. The final clones should originate from different *Col1a1-frt* targeted parental clones to minimize the chance of miss-interpreting phenotypes due to possible unwanted genetic alterations introduced by long-term culture. The selection of best-performing *Col1a1-frt* targeted GEMM-ESC clones is crucial for the efficiency to later generate experimental cohorts as the number of chimeras born per injected embryo is likely to decline after additional manipulations and propagation in culture.

The GEMM-ESC procedure as presented here, can be divided in two separate phases: a resource phase and an experimental phase (Fig 5). The resource phase, starting from the selection of the original GEMM until cryogenic storage of quality controlled *Col1a1-frt* targeted GEMM-ESC clones is fairly laborious, taking up to 6 months. The success rate is high and independent of the strain background. This can be largely attributed to the optimized ESC culture conditions, which not only simplify procedures but also allow for better quality ESCs as compared to previous protocols. The 2i culture protocol also enables derivation of ESCs from mouse strains that were previously considered refractory (Ying *et al*, 2008; Reinholdt *et al*, 2012). In this study, we derived 47 ESC clones from 364 embryos, representing 13% derivation efficiency. Although this seems low, most of our ESC derivation attempts were successful. The derivation efficiency could be further increased by expanding all early ESC clones instead of selecting them on the basis of their morphology and growth rate (Table 1).

One issue we noted is that different genetic backgrounds of the original GEMMs require fine-tuning of the ESC injection procedure in order to achieve an optimal balance between quality and yield of the resulting chimeras. This balance is affected by several factors, including the strain background and the developmental stage of the host embryos. Injection of ESCs into morulae instead of blastocysts (Plagge *et al*, 2000) leads to very high quality chimeras but also causes an increase in birth problems, still-born animals and pups with growth retardation, in particular for FVB/n and FVB/n;129/Ola ESC clones (Fig 1D and supplementary Table S1). We therefore optimized the injection procedure for each ESC background. In practice, ESCs derived from C57BL/6J strains were injected into FVB/n morulae, whereas ESCs from FVB/n;129/Ola or FVB/n strains were injected into C57BL/6N blastocysts. Also other strategies are available to shift the balance between the quality and the yield of chimeras. For instance, fully ESC-derived chimeras can be produced using tetraploid complementation techniques (Eakin & Hadjantonakis, 2006). Alternatively, lower numbers of ESCs can be injected per morula or blastocyst in order to improve the yield of life-born chimeras.

The quality of GEMM-ESC clones remains stable over time, even after genetic manipulation by targeting or Flp-mediated integration, as 70–78% of newly modified ESC clones gave good chimeras and GLT (Fig 5). The genetic stability of ESCs cultured in 2i medium is comparable to ESCs cultured under classic conditions (Fig 3B; Liang *et al*, 2008). The occurrence of CNVs highlights the need for thorough genetic screening of the *Col1a1-frt* targeted GEMM-ESC clones by e.g. aCGH, deep sequencing or spectral karyotyping, especially because a fraction of these CNVs will be transmitted from the chimeras to the F1 offspring (Fig 3C). The development of CNVs might be related to targeting or Flp-mediated integration; however, we have also observed CNVs in freshly subcloned ESCs (supplementary Table S4). It is therefore more likely that CNVs arise spontaneously when cells are stressed, for instance by single-cell cloning or antibiotic selection regimens, and not by specific recombination or integration events. Occasionally, we observed identical CNVs in independent subclones from the same GEMM-ESC, implying that some CNVs are already present in a subpopulation of the derived GEMM-ESC clone (supplementary Table S4). This places the occurrence of some CNVs very early in the ESC derivation process. Thus far, we did not observe any phenotypic changes in our chimeras or their offspring that could be attributed to a particular CNV; however, this remains a possibility. Also, aCGH screening may not allow for full identification of all aberrations that occur during *in vitro* culture as some apparently normal clones failed to give rise to chimeras indicating an underlying genetic or epigenetic defect. It should be noted that these issues are not specific for the approach described here but plays a role in any experiment using cloned ESCs (Liang *et al*, 2008); in a conventional ESC approach these CNVs are likely bred out of the cohort which is not the case when chimeras are used directly, although use of independent ESC clones can further reduce this risk. Further refinements in the ESC culture conditions might improve the (epi)genetic stability of the GEMM-ESC clones.

The experimental phase of the GEMM-ESC procedure is based on the ‘plug-and-play’ principle, which ensures optimal flexibility and short time frames. It takes typically <4 months to obtain an experimental cohort. Another advantage of the plug-and-play system is that it is compatible with a wide array of vectors for gain-of-function or loss-of-function studies. To this end, we are developing additional Flp-in vector backbones for inducible expression of shRNAs to allow for specific knockdown of target genes. In the near future one only has to clone a cDNA or validated shRNA construct into one of these vectors and introduce that into the GEMM-ESC clone of choice after which an experimental cohort can be produced. This will permit the swift validation of candidate cancer genes identified in genomic sequencing efforts and functional genetic screens in a suitable mouse model, as we have shown for Mycl1. Moreover, in the case where full target gene silencing is required to observe a phenotype, the highly efficient gene editing tools, TALENS and CRISPR/Cas, can be applied on GEMM-ESCs to generate a homozygous null allele that can be directly evaluated in chimeras (Fig 5). By using a GEMM instead of a xenograft model for *in vivo* validation, also the effects of the target gene on tumor initiation, tumor progression and tumor maintenance can be monitored, including interactions between tumor cells and immune cells.

The GEMM-ESC procedure allows for the direct use of chimeras for experimentation. We show that the tumor leftacteristics of chimeric mice are very similar to those of conventional mice (Fig 2), though the tumor latency can differ depending on the model and level of chimerism (Fig 4C, supplementary Fig S2). Typically, the level of chimerism is estimated on basis of coat-color contribution, although this consistently results in an overestimation of the true chimerism in the various tissues (Fig 2C and D). In our experience 70–100% chimeras give a quite consistent reproducible tumor phenotype when the penetrance is high. In GEMMs with low penetrant phenotypes it is advisable to backcross the chimeras to the parental strain and use the F1 cohort. We feel that a more quantitative analysis on a particular tissue, e.g. tail, does not provide a substantial advantage above estimating chimerism on the basis of coat color, as variations can also be found among different tissues. The chimeric approach is particularly useful for side-by-side comparison of multiple allelic variants of the same gene, or for *in vivo* screening of multiple candidate cancer genes in one GEMM in a semi-high throughput fashion. Direct comparison of tumorigenesis in chimeras that differ only on basis of the expression of one candidate cancer gene will accelerate the identification of true driver genes within a large group of candidate cancer genes, for instance *Mycl1* in the SCLC model. Furthermore, all tumors will have an identical genetic background, excluding any variation caused by modifier alleles introduced via breeding. This simplifies subsequent deep sequencing efforts as all tumors can be compared to the same GEMM-ESC derived reference DNA. It is, however, advisable to use two independent ESC lines as also recombinase-mediated introduction of constructs into the ESCs can give rise to chromosomal changes that could influence the outcome.

We realize that in most cases the chimeras will be crossed to the original GEMM to establish stable mouse strains. Indeed, a single cross of the chimeras to the original strain will result in F1 mice with or without the introduced construct. These mice can be directly used as experimental and control cohorts, respectively (Fig 4D). The feasibility of this approach depends on the efficiency of GLT of the chimeras. Based on two GEMMs we observed that twelve out of fourteen chimeras gave GLT in their first litter and one chimera in the second (supplementary Table S1). Furthermore, 40% of the F1 mice contained the construct, reflecting a very efficient transmission. Compared to the classic approach, where many crosses are required to obtain a particular genotype, the F1 approach still provides a considerable time gain since it only adds ˜10 weeks to the chimeric GEMM-ESC approach.

In conclusion, the GEMM-ESC method performs well on all fronts and can be routinely applied in transgenic facilities to accelerate the generation and adaptation of mouse models. Our GEMM-ESC clones will be distributed via the European Mouse Mutant Archive (http://www.emmanet.org/), which is part of the INFRAFRONTIER Research Infrastructure (http://www.infrafrontier.eu), to make mouse models of human cancer more accessible to the scientific community.

## Materials and Methods

### Ethics statement

The study was performed in accordance with the Dutch and European regulations on care and protection of laboratory animals. Mice were housed under standard conditions of feeding, light and temperature with free access to food and water. All animal experiments have been approved by the local animal experimental committee, DEC NKI (OZP ID: 10023).

### Mouse strains

*Kras*^*LSL-G12D*^ mice, *Rb1*^*F/F*^ *;Trp53*^*F/F*^ mice and *Nf2*^*F/F*^ *;Trp53*^*F/F*^ ;Cdkn2a^*/*^ mice have been described earlier (Jackson *et al*, 2001; Meuwissen *et al*, 2003; Jongsma *et al*, 2008). Wild-type FVB/n, C57BL/6N and B6CBAF1/Ola mice were purchased from Harlan, and wild-type C57BL/6J mice from Leftles River.

### Embryonic stem cell derivation and culture

Eight-cell stage embryos or morulae were flushed from the oviduct of superovulated females at 2.5 days *post coïtum*. Derivation of ESCs was performed according to the protocol described by Nichols *et al* (2009). ESCs were cultured on 0.1% gelatin-coated plates in N2B27 medium (Ying *et al*, 2003; DMEM/F12 from Gibco, Neurobasal medium, N2 supplement and B27 supplement from Invitrogen) with LIF (Chemicon) and 2i, i.e. 1 μM PD0325901 (Axon Medchem) and 3 μM CHIR99021 (Axon MedChem). ESCs were split between 1:4 and 1:8 every 2–3 days and dissociated with Accutase (Sigma). Cells were cultured at 37°C in 5% CO2. All primary ESC cultures were tested negative for specific mouse pathogens in a MAP PCR test performed by QM Diagnostics, Nijmegen, the Netherlands. The gender of the derived ESCs was determined by PCRs specific for the X-chromosome, by detection of gene Uba1 with primers 5′-TGGTCTGGACCCAAACGCTGTCCACA-3′ and 5′-GGCAGCAGCCATCACATAATCCAGATG-3′, product size 210 bp, and the Y-chromosome, by detection of gene Sry with primers 5′-CCCCATGAATGCATTTATGGTGTGGT-3′ and 5′-CTTGCCTGTATGTGATGGCATGTGGG-3′, product size 326 bp. IB10 cells (Robanus-Maandag *et al*, 1998) were maintained on irradiated mouse embryonic fibroblasts in GMEM (Invitrogen), β-mercaptoethanol (Sigma), fetal bovine serum (Hycult) and LIF and weaned towards 2i conditions by culturing the cells for several days in a hybrid medium of 50% GMEM+β-me+FBS+LIF and 50% N2B27+2i+LIF on 0.1% gelatin-coated plates.

### Injection of embryonic stem cells into embryos

Male ESCs (12–15 cells) from FVB/n or FVB/n;129/Ola strains were injected into C57BL/6N blastocysts as described (Hogan *et al*, 1994) and implanted into pseudopregnant B6CBAF1/Ola fosters. For morulae injection, 4–8 male ESCs from the C57BL/6J strain were injected under the zona pellucida of an FVB/n embryo (Plagge *et al*, 2000). Following injection, the chimeric embryos were cultured overnight in KSOM medium (Chemicon) to blastocyst stage and implanted in B6CBAF1/Ola fosters. We aimed for 3–6 chimeras, corresponding to 30–60 implanted embryos per ESC injection session. The percentage of chimerism of the resulting chimeras was scored by two researchers (by IH and RBA) based on the absence of host derived coat color. Germline transmission was determined by crossing male chimeric mice to FVB/n females and the offspring were scored for either coat color transmission or presence of the mutant allele as detected by PCR.

### Flow cytometry for intracellular stem cell markers

Single cell suspensions of ESCs were fixed and permeabilized using the Foxp3 staining buffer set (eBioscience). Aspecific binding was prevented by blocking for 15 min at 4°C with isotype controls, i.e. 0.125 μg/ml rat IgG2b (14-4031; eBioscience), 0.5 μg/ml rat IgG 2aΚ (16-4321; eBioscience) and 0.05 μg/ml mouse IgG2a (MAB003; R&D systems). Staining was performed in the dark for 30 min at 4°C with 0.125 μg/ml anti-human/mouse Oct3/4-PE (IC1759P; R&D systems), 0.5 μg/ml anti-mouse Nanog-488 (53-5761; eBioscience) and 0.05 μg/ml anti-human/mouse Sox2-APC (IC2018A; R&D systems). Cells were washed twice in 1× permeabilization buffer and resuspended in PBS with 0.5% bovine serum albumin. Fluorescence was measured on the FACScalibur (BD Biosciences) and analyzed with the FlowJo software (version 8.8.7).

### Flp-in targeting construct and Luciferase reporter constructs

The *Col1a1-frt* targeting construct and both the Flpe and GFP overexpression plasmids were kindly provided by J. Gribnau, Erasmus Medical Left Rotterdam (Beard *et al*, 2006). The *frt-invCAG-Luc* and *frt-invEF1-Luc* vectors were identical apart from the promoter sequence used, which was either the chicken β–actin (CAG) promoter or the EF1a promoter, respectively. The constitutive promoter was followed by a lox71 site, an ATG-coding Frt site, a firefly Luciferase2 ( *Luc*) gene with polyadenylation site in the opposite orientation, and a lox66 site followed by three modules of splice acceptor:polyadenylation site also all in the opposite orientation of the promoter sequence. In the *frt-invCag-MycL1-Luc* vector, the *MycL1* cDNA and IRES were place between the ATG-coding Frt site and the *Luc* gene of vector *frt-invCag-Luc*. After Flp-mediated integration of these vectors in the *Col1a1* locus they act as inversion transgenes that display conditional expression of the Luciferase gene after Cre recombination.

### Genetic engineering of GEMM-ESCs under 2i culture conditions

GEMM-ESCs (5 × 10^6^) were electroporated with 10 μg XhoI digested *Col1a1-frt* targeting plasmid DNA in 0.4 cm Gene Pulser cuvettes (Biorad) at 3 μF, 0.8 kV for 0.1 ms in a Biorad Gene Pulser. Cells were plated on a 57 cm^2^ tissue culture dish pre-coated with 0.1 mg/ml laminin (Sigma). After 24 h antibiotic selection was started with 200 μg/ml Geneticin (Gibco) in N2B27+2i+LIF. The medium was refreshed every other day. After 10–14 days individual clones could be picked, dissociated and transferred to a 0.1% gelatin-coated 96-well plate in N2B27+2i+LIF. Once ESC spheres reached subconfluency, they were resuspended to detach from the plate (spheres are only loosely attached) and half of the volume was used for genomic DNA extraction of detached spheres. PCR screening was performed on the same day. Positive ESC clones were expanded for both cryopreservation and for genomic DNA extraction to perform additional quality controls, such as Southern blot analysis and aCGH.

Flp-in was achieved by co-transfecting three plasmids in a *Col1a1-frt* targeted GEMM-ESC clone, one plasmid expressing Flp^e^ (0.6 μg) one expressing GFP (0.6 μg) and the Flp-in vector, either *frt-invCAG-Luc*, *frt-invEF1-Luc* or *frt-invCag-MycL1-Luc* (4.8 μg). Two days in advance 1 × 10^6^ cells were seeded on a laminin-coated 57 cm^2^ dish. Plasmid DNA diluted in 250 μl OptiMEM I reduced serum medium (Gibco) was mixed with 9 μl Lipofectamin 2000 (Invitrogen) diluted in 250 μl OptiMEM, mixed gently and incubated for 20 min at room temperature. A mixture of DNA:Lipofectamin (500 μl) was added to the culture dish containing the *Col1a1-frt* GEMM-ESC clone and gently rocked. After 6 h the medium was replaced for fresh N2B27+2i+LIF. Transfection efficiency was evaluated the next day by monitoring for green fluorescence. After 24 h, Hygromycin-B (150 μg/ml; Invitrogen) was added and medium was refreshed every other day. Again individual clones were visible and picked after 10–14 days. Subsequent culture and processing was similar as described for the targeted clones.

### PCR screening of targeted and Flp-in ESC clones

The same PCR was used for screening of the *frt-Col1a1* targeted ESC clones and determining GLT of the mutant allele in the offspring of the chimeric mice. The forward primer was located in the Hygromycin-B gene, 5′-GCCCCAGCACTCGTCCGAGGGC-3′, and the reverse primer in the *Col1a1* locus downstream of the right homology arm present in the targeting vector, 5′-CCCAGGTCCTGCCTCCTCCGTGC-3′. Both cells and tails were lysed in DirectPCR lysis reagent (Viagen Biotech) with 0.25 mg/ml Proteinase K (Sigma) at 55°C. PCRs were performed in 1x Phusion Flash high-fidelity PCR mix (Thermo Scientific) with a 71°C annealing temperature and an 80 s elongation time. Product size was 3.0 Kb. Screening of *frt-invCAG-Luc* and *frt-invCag-MycL1-Luc* Flp-in ESC clones was performed with a forward primer located in the CAG promoter, 5′-CTGCATCAGGTCGGAGACGCTGTCG-3′ and the reverse primer in the Hygromycin-B gene, 5′-GGGTTCGGCTTCTGGCGTGTGACC-3′. Product size was 319 bp. Screening of *frt-invEF1-Luc* Flp-in ESC clones was performed with primers located in the *Luc* gene, with forward primer 5′-CTTCGAGGAGGAGCTATTCTTGCG-3′ and reverse primer, 5′-CTGGTAGGTGGAAGCGTTTGGC-3′. Product size was 203 bp.

### Southern blot analysis

Genomic DNA from ESCs and organs was extracted with the Gentra Puregene Tissue Kit (Qiagen). Genetic chimerism in various tissues was determined by Southern blotting of EcoRV digested DNA hybridized to the *Trp53* 5′ XbaI probe, which is a 700 bp genomic XbaI fragment subcloned in pBSK and labeled by PCR (Jonkers *et al*, 2001). Intensity of bands was quantified with ImageJ software (version 1.43u) by generating a profile plot and measuring the surface area of individual peaks. Targeting with the *Col1a1-frt* vector was monitored by Southern blotting of EcoRI digested DNA hybridized to the *Col1a1* 3′ probe, which is an 842 bp genomic PstI-XbaI fragment. Flp-in of *frt-invCAG-Luc* and *frt-invEF1-Luc* was monitored by Southern blotting of BglII digested DNA hybridized to the same *Col1a1* 3′ probe.

### Ad5-Cre virus administration

Mice were treated with cyclosporine A (Novartis) orally in the drinking water, 1 week prior to adenovirus administration and 2–3 weeks following infection. Viral Ad5-CMV-Cre particles (1 × 10^9^; Gene Transfer Vector Core, University of Iowa) were injected intratracheally into *Kras*^*LSL-G12D*^ mice and *Rb1*^*F/F*^ *;Trp53*^*F/F*^ mice and intrathoracically into *Nf2*^*F/F*^ *;Trp53*^*F/F*^ ;Cdkn2a^*/*^ mice.

### Immunohistochemistry of tumors

Mice were monitored biweekly for development of tumors and general health status. Mice were sacrificed when signs of discomfort became evident. Tissues were collected for pathological examination. Formalin fixed and paraffin embedded material was sectioned, H&E stained an analyzed microscopically by a dedicated mouse pathologist (by JYC).

### Array comparative genome hybridization

Genomic DNA from ESCs was extracted with the Gentra Puregene Tissue Kit (Qiagen). DNA (500 ng) was labeled with either Cy5 or Cy3 using the NimbleGen Dual-Color DNA Labeling Kit (NimbleGen). Labeled DNA was hybridized on the mouse array comparative genome hybridization (aCGH) 12 × 135 K whole-genome tiling array (NimbleGen) using the MAUI hybridization station. Targeted ESC clones were analyzed against the parental ESC clones and the Flp-in ESC clones against the targeted ESC clones. Data was analyzed using the NimbleScan (Roche) and Nexus 6.0 (BioDiscovery) software. Data are available at the MIAMExpress database (http://www.ebi.ac.uk/miamexpress) under accession number E-MEXP-3998.

**The paper explained**

**Problem**

The ever-growing list of potential cancer genes and drug targets associated with specific cancers demand validation in relevant *in vivo* models. Genetically Engineered Mouse Models (GEMMs) have proven valuable for the distinction of driver mutations from less relevant modifiers or bystanders, though the inherent complexity of these models and time spent introducing additional modifications has hampered their general use.

**Results**

We have developed an approach for fast and flexible adaption of existing mouse models. Three elements are central to this system; (i) The efficient derivation of authentic Embryonic Stem Cells (ESCs) from established GEMMs, (ii) the routine introduction of transgenes of choice in these GEMM-ESCs by Flp recombinase-mediated integration and (iii) the direct use of the chimeric animals in tumor cohorts. By applying stringent quality controls, the GEMM-ESC approach proofs to be a reliable and effective method to speed up cancer gene assessment and target validation, as exemplified for MycL1 in a model for Small Cell Lung Cancer.

**Impact**

The GEMM-ESC approach speeds up the generation/modification of mouse models, while minimizing the breeding efforts. Experimental cohorts of mice are generated on-demand and ready-to-use. This reduces the number of mice needed per experiment and is therefore in compliance with one of the 3R's of animal testing. Furthermore, the establishment of an archive of ESCs from various validated mouse models will allow easy distribution and gives researchers worldwide the opportunity to evaluate their favorite genes in the most suitable model.

### DNA copy number analysis

Genomic DNA from tumors was extracted with the Gentra Puregene Tissue Kit. Real time PCR was performed on genomic DNA using SYBRGreen in the StepOnePlus real time PCR system (Applied Biosystems) with primers specific for MycL1 (Dooley *et al*, 2011) and related to at least two reference genes in the same sample, either Actin, GapdH, Tfrc or Tert. Primer sequences are provided in supplementary Table S5. Some samples were analyzed by low-coverage sequencing. Nexus 6.0 software was used to process Illumina Hiseq2000 generated signal intensity. A reference samples was created by combining three ESC clones, *invCag-Luc;Rb1*^*F/F*^ *;Trp53*^*F/F*^ clones 1.5_1B1_6 and _ *9* and *Col1a1-frt;Rb1*^*F/F*^ *;Trp53*^*F/*F^ clone 1.5_1B1_r4. The FASST2 segmentation and default Illumina setting, with Gain 0.4 and Loss −0.4, were used to identify regions of CNV for each sample.

### *In vivo* bioluminescence imaging of tumors

*In vivo* bioluminescence imaging was performed and quantified as described by Hsieh *et al* (2005) on a cryogenically cooled IVIS system (Xenogen Corp., CA, USA) using LivingImaging acquisition and analysis software (Xenogen).

### Statistical analysis

Survival analysis was performed by comparing two survival curves using the Log-rank (Mantel-Cox) test within the Prism 6 software. The comparison of the incidence of MycL1 copy number gains between three groups was performed using the Fisher's Exact Test. The cut-off for MycL1 amplified tumors was more than four copy numbers present in the tumor.

## For more information

Website: http://www.mccanet.nl
