# Supplementary material for: Rapid target gene validation in complex cancer mouse models using re-derived embryonic stem cells
Source: EMBO Mol Med. 2014 Jan 15;6(2):212–25. doi: 10.1002/emmm.201303297 (PMC3927956; doi:10.1002/emmm.201303297)
Supplement: Supplementary file 1 [file emmm0006-0212-sd1.pdf]

EMBO Molecular Medicine

Rapid target gene validation in complex cancer mouse models using re-derived embryonic stem cells

Ivo J Huijbers, Rahmen Bin Ali, Colin Pritchard, Miranda Cozijnsen, Min-chul Kwon, Natalie Proost, Ji-Ying Song, Hilda de Vries, Jitendra Badhai, Kate Sutherland, Paul Krimpenfort, Ewa M Michalak, Jos Jonkers & Anton Berns

Corresponding author: Anton Berns, Netherlands Cancer Institute

Review timeline:

Submission date:	18 July 2013
Editorial Decision:	28 August 2013
Revision received:	27 September 2013
Editorial Decision:	14 October 2013
Revision received:	22 October 2013
Accepted:	22 October 2013

Transaction Report:

(Note: With the exception of the correction of typographical or spelling errors that could be a source of ambiguity, letters and reports are not edited. The original formatting of letters and referee reports may not be reflected in this compilation.)

Editor: Céline Carret

1st Editorial Decision 28 August 2013

Thank you for the submission of your manuscript to EMBO Molecular Medicine. We have now heard back from the three referees whom we asked to evaluate your manuscript. As you will see, all three referees are enthusiastic about the paper and mainly ask for further details and clarifications of the findings.

Given the balance of these evaluations, we I will be happy to consider a revision of your manuscript if you can address the issues that have been raised to the satisfaction of the referees. Please note that it is EMBO Molecular Medicine policy to allow a single round of revision in order to avoid the delayed publication of research findings. Consequently, acceptance or rejection of the manuscript will depend on the completeness of your responses included in the next version of the manuscript.

I look forward to seeing a revised form of your manuscript as soon as possible.

***** Reviewer's comments *****

Referee #1 (Comments on Novelty/Model System):

As clearly explained by the authors in the introduction, the increasing complexity of genetically engineered mouse models used to mimic human diseases, is becoming a problem to rapidly test new candidate genes. Previously the authors and others have shown that embryonic stem (ES) cells derived from these strains can be used to speed-up this process. In the current manuscript, the

authors present optimized methods for both the generation of these ES cells, validation of their quality, as well as their application to test the importance of a candidate gene, in this case MycL1. Both the technical quality as well as the validity of the performed experiments are of high quality. The general strategy is however not entirely novel and builds on previous publications of themselves as well as others, hence the medium score for novelty. In addition, the manuscript is largely a descriptive manuscript of the technical requirements to use this technique, and as such will mainly be of interest to researchers that wish to adopt this technique in their research. Nevertheless, the general approach is likely becoming an important method in the future, thereby complementing current methods, and as such is likely to impact upon medical research. Overall, I believe this manuscript to be suitable for publication in EMBO Molecular Medicine after some minor modifications.

Referee #1 (Remarks):

The manuscript by Huijbers et al describes the use of re-derived embryonic stem cells for target gene validation in genetically complex mouse models. This methodology has been developed by independent groups several years ago, and was also proposed for tumor models by the authors themselves. In the current manuscript they describe a streamlined protocol for two lung tumor models and one mesothelioma model. It provides a fair and balanced description of both the advantages as well as the obstacles that have to be taken to establish this technique for a specific GEMM. As such, it represents an important paper for other researchers that wish to adopt this technique within their institute. In addition, they use this technique to confirm that MycL1 is an important driver gene in small cell lung cancer. Overall, the manuscript is well written and of technically high quality.

Other remarks:

- On page 8 it is stated that the coat-color chimerism well reflects the genetic chimerism. However, Figure 2C and 2D show various examples that this is not the case, as several animals show genetic chimerism, which is 50-60% lower than scored by coat-color. Clearly, coat-color is not a reliable indicator of true chimerism, while nevertheless it is used throughout the manuscript. I would suggest the authors to propose a more reliable method for chimerism determination, e.g. southern blot on tail DNA, as for other GEMMs with a less penetrant phenotype, a low chimerism may represent a problem.
- Judging from table S1, the number of chimeras born following embryo injection can be highly variable, and is occasionally very low. Do the authors have a suggestion how to deal with this in practice when generating experimental cohorts?
- I am not familiar with the meaning of the "*" used in Cdkn2a*/*. Please, provide a brief explanation.
- It took me a while to understand the meaning of the numbers "3.2", "4.1", and so on, in Figure 3C. Later it became clear to me that this refers to the numbers in Table S3. I would suggest to refer to this in the figure legend.

Referee #3 (Remarks):

The manuscript by Huijbers et. al describes the generation and validation of a method for generating mouse cancer models directly from embryonic stem cells. Overall the manuscript is relatively clear and well written and the data is clearly presented, and it describes in detail, an approach which will be of significant interest to the readers of the journal. There are however a number of issues which would require to be addressed before publication.

Figure 1A. Although these data may well be of some interest to scientists working in this specific area, it is simply reproducing the data previously described in Ying et al, and subsequent publications on this topic with unmodified wild-type cells. As a consequence this data should be

replaced with the data relating to the cell lines generated for this study or removed from the manuscript. Figure 1B-E, the differences between morula and blastocyst injection and already well established. This data is again generated from wild-type cells and I don't see the point of reproducing it here, especially when the majority of the animals discussed in this study (including those generated from all of the targeted ES cell lines) were generated by blastocyst injection.

Figure 2 C and D are unnecessarily complicated and detract from the message behind the data. The comparison of coat colour to genetic contribution in each tissue simply informs how efficient the authors are at estimating contribution by coat colour. This data could simply be replaced by a more simple analysis which estimates the average contribution in chimeras 70% and above in each tissue or something similar.

Figure 2 E and F and supporting information 2. It is quite clear there is a significant difference in the survival curve in the Kras-LSLG12D chimeras. It is not clear why this is in the supplementary data rather than the actual manuscript. The most likely explanation for this is that the ES cells have undergone a genetic change during the culture process, which is causing this reduction in survival. This should be discussed by the authors, as it is obviously highly relevant to the efficiency of this approach.

The authors analyse copy number variation in the targeted cell lines, but do not do so in the originally derived ES cell lines. It would be useful for them to do so as it may be that CNVs are observed equally frequently during the process of ES cell derivation. This may also explain the difference in the survival curve seen in the Kras chimeras.

Figure 3B should include the actual number of cell lines which are normal and those with CNVs, as it is hard to work out the actual figures from this graphical representation. In figure 3C, 2 of the 3 cell lines analysed contain CNVs. The 3rd 'normal' cell line generated no chimeras from 88 injected blastocysts, suggesting this cell line is also carrying an undetected genetic alteration. Consequently in this analysis, these data imply that the vast majority of targeted cell lines (100%) are carrying genetic alterations. This is completely at variance with the way the data is described in the rest of the manuscript (e.g. figure 3b) and is obviously highly relevant to the efficiency with which this approach can be used. Again this should be highlighted and discussed by the authors.

The data generated in figure 4C comparing the survival curve of chimeras expressing the MycL1 cDNA with that of controls, contracts with the data derived from the F1 animals. Again this represents a big potential limitation of the system the authors describe as it clearly shows that significant differences generated in genetically modified animals cannot be replicated in chimeras. This issue should be addressed by the authors. In figure 4F, although apparently not statistically significant, there is a difference in percentage terms between the Rb;Trp53 mice and the Rb;Trp53;inv-Cag-Luc mice which are supposed to be a negative control. The reason behind this large difference should be addressed.

It appears that there is a consistent reduction of the percentage chimerism obtained at each stage of the process (compare figure 2A and B with figure 3A), although this does not appear to effect germline transmission. Again this may have a big effect on the utility of this approach and should be discussed by the authors. It also implies that there is a limit to the number of modifications which could be made to each

The authors suggest that this approach would be frequently used to generate F1 cohorts of mice. The author's should probably point out that this would considerably reduce the range of applications of this technology as it does not allow homozygous KO animals to be analysed.

Also they make reference to using this system for genetic screen. The observed number of CNVs would suggest that the false positive rate would be be extremely high using such an approach. This should be discussed in the text.

Minor points. I don't really see how this can be described as a pipeline, so reference to this should be removed and replaced by a more suitable word such a methodology or approach.

In the panels relating to FACS analysis there are cell populations in blue and red. I am assuming the red population represent the isotype control but this should be clearly stated in the manuscript.

Referee #4 (Remarks):

Genetically engineered mouse models (GEMMs) can provide important insights into the molecular basis of tumor development and enable testing of new intervention strategies, but their inherent complexity, combined with the time and expense it takes to development these models, has hampered their use as preclinical models for validating cancer genes and drug targets. In an attempt to accelerate target gene validation in mouse models the authors have developed an alternative strategy, which involves re-deriving embryonic stem cells (ESCs) from well-established and validated GEMMs. These GEMM-ESCs are then used for further genetic engineering either by classic gene targeting, gene editing or recombinase-mediated transgene integration. These GEMM-ESCs contain the same genetic modifications present in the original model plus the newly introduced genetic modification, such as the conditional overexpression of an oncogene or the conditional loss-of-expression of a tumor suppressor gene. The modified GEMM-ESCs can then be used to generate high quality chimeras that are likely to be equally susceptible to tumor induction as the original GEMM. The main advantage of this approach is its speed and flexibility, which makes it possible to comparatively analyze the phenotypic consequences of different genes and allelic series in a particular GEMM. Instead of crossing the chimeric mice to the desired strain and genetic background, ready-to-use GEMMS can now be produced on demand, which reduces the cost and total number of mice needed per experiment. This approach also allows for the establishment of a GEMM-ESC bank for the distribution ESCs with complex genotypes.

The feasibility of the GEMM-ESC production pipeline depends on reliable procedures and robust quality controls. In the experiments described here the authors have developed and validated such a pipeline for three GEMMs: two lung cancer models and one mesothelioma model. First, they developed and validated an efficient method for deriving ESCs from established GEMMs. Next, they introduced a Flp-in module just after the 3' UTR of the Col1a1 locus. This module serves as a docking sites, which permits the introduction of oncogenes or shRNAs for down-regulating tumor suppressor genes by Flp recombinase-mediated integration. This makes it possible to rapidly re-engineer the GEMM-ESCs by Flp-mediated recombination. Finally, they used one of their GEMM-ESC models to validate Mycl1 as a bona fide oncogene in small cell lung cancer.

Overall, this is a well done and through study, which provides compelling evidence supporting the use of GEMM-ESCs for rapid and high-throughput candidate cancer gene assessment and validation. Over the past few years literally hundreds of new candidate cancer genes have been identified by cancer genome resequencing or through the use of mouse models. However, due to the shear number of genes being identified, classical methods for target validation, which include conventional transgenic and mouse knockouts, are not realistic. Faster and less costly methods like the one described here need to be developed. The one drawback of the method described here is the time it takes and the specialized expertise needed to make new GEMM-ESC models. Fortunately, these models can be easily distributed once they are made through distribution centers, such as the European Mouse Mutant Archive, once experts in the field generate them. In this regard, it is important to note that all three GEMM-ESC models described here will be made freely available through the European Mouse Mutant Archive. These models will no doubt be of great value to people who wish to validate candidate cancer genes identified in lung and mesothelioma cancer resequencing studies. While this manuscript is largely suitable for publication in EMBO Molecular

Medicine in its present form there are a few minor issues that that authors need to address before publication.

Review Comments:

1. The authors need to more fully describe the rationale for using the Flp-in module and why it was targeted to the 3' UTR of Col1a1. There are other regions in the mouse genome where they could have targeted this module, such as Rosa26. Why was this particular locus picked? Also, why did they choose to use this module and not another module that uses for example Cre or some other docking site such as a lambda attachment site?
2. The correct strain designation is C57BL/6J and not C57Bl6J.
3. What is meant by Cdkn2a*/*? What do the *'s mean? The authors should use the correct locus designation to describe this mutant gene.
4. In the Discussion the authors state that an ESC coat-color contribution of at least 35% is required for efficient tumor induction studies. On what basis do they make this statement? It seems like the contribution required will dependent on how much the introduced oncogene or shRNA reduces the tumor latency. Some oncogenes or tumor suppressor genes might have a large effect on tumor latency while other might have a weaker effect. The required coat-color contribution might thus vary depending upon the gene being validated. The authors should comments on this in the revised paper.

1st Revision - authors' response 27 September 2013

Referee #1 (Remarks):

The manuscript by Huijbers et al describes the use of re-derived embryonic stem cells for target gene validation in genetically complex mouse models. This methodology has been developed by independent groups several years ago, and was also proposed for tumour models by the authors themselves. In the current manuscript they describe a streamlined protocol for two lung tumour models and one mesothelioma model. It provides a fair and balanced description of both the advantages as well as the obstacles that have to be taken to establish this technique for a specific GEMM. As such, it represents an important paper for other researchers that wish to adopt this technique within their institute. In addition, they use this technique to confirm that MycL1 is an important driver gene in small cell lung cancer. Overall, the manuscript is well written and of technically high quality.

Other remarks:

- On page 8 it is stated that the coat-colour chimerism well reflects the genetic chimerism. However, Figure 2C and 2D show various examples that this is not the case, as several animals show genetic chimerism, which is 50-60% lower than scored by coat-colour. Clearly, coat-colour is not a reliable indicator of true chimerism, while nevertheless it is used throughout the manuscript. I would suggest the authors to propose a more reliable method for chimerism determination, e.g. southern blot on tail DNA, as for other GEMMs with a less penetrant phenotype, a low chimerism may represent a problem.

From page 8 we removed the sentence: “The overall percentage of genetic chimerism was well reflected by the coat-colour chimerism”. In the discussion we added the following sentences: Typically, the level of chimerism is estimated on basis of coat-colour contribution, although this consistently results in an overestimation of the true chimerism in the various tissues (Fig 2C-D). In our experience 70-100% chimeras give a quite consistent reproducible tumour phenotype when the penetrance is high. In GEMMs with low penetrant phenotypes it is advisable to backcross the chimeras to the parental strain and use the F1 cohort. We feel that a more quantitative analysis on a particular tissue, e.g. tail, does not provide a substantial advantage above estimating chimerism on the basis of coat colour, as variations can also be found among different tissues.

- Judging from table S1, the number of chimeras born following embryo injection can be highly variable, and is occasionally very low. Do the authors have a suggestion how to deal with this in practice when generating experimental cohorts?

We are aware of this variability. We are taking active steps to address these issues, some of which are mentioned in the manuscript:

(1) Further optimize injection procedures. In the manuscript we describe our current status, but we are exploring options for further improvements. For instance, we are trying to identify better-suited host embryos from other strain backgrounds.

(2) Screen several *Col1a1-frt* targeted GEMM-ESC clones to identify best performing clones and use those for the experimental phase. In the legend of Fig 5 we included a practical guideline:

"In practice, we advise that for each model (i) multiple *Col1a1-frt* targeted GEMM-ESC clones are screened for their ability to efficiently generate high quality chimeras, (ii) two of the best-performing clones are selected for the Flp-in procedure, and (iii) at least two transgene-coding GEMM-ESC clones are used to generate cohorts. The final clones should originate from different *Col1a1-frt* targeted parental clones to minimize the chance of miss-interpreting phenotypes due to possible unwanted genetic alterations introduced by long-term culture. The selection of best-performing *Col1a1-frt* targeted GEMM-ESC clones is crucial for the efficiency to later generate experimental cohorts as the number of chimeras born per injected embryo is likely to decline after additional manipulations and propagation in culture."

(3) Increase micro-injection capacity. In the last three years we have increased our injection capacity by 2 to 3-fold. The practical guideline and the experimental details described in our manuscript should enable ESC/transgenic facilities to successfully implement the GEMM-ESC approach and to successfully produce experimental cohorts.

- I am not familiar with the meaning of the "" used in Cdkn2a*/*. Please, provide a brief explanation.*

The Cdkn2a* allele contains a point mutation in p16^{Ink4a} at conserved amino-acid position 101 that results in a stop codon thereby creating an unstable protein and functional loss of p16^{Ink4a}, while leaving the alternative reading frame protein p19^{Arf} unaffected. At the top of page 7 a clarification is provided in the text including a reference to the original paper describing this mutant mouse strain.

- It took me a while to understand the meaning of the numbers "3.2", "4.1", and so on, in Figure 3C. Later it became clear to me that this refers to the numbers in Table S3. I would suggest to refer to this in the figure legend.

The following sentence is added to the legend of Figure 3C: "A detailed description of all CNVs is provided in Supporting Information Table 3".

Referee #3 (Remarks):

The manuscript by Huijbers et. al describes the generation and validation of a method for generating mouse cancer models directly from embryonic stem cells. Overall the manuscript is relatively clear and well written and the data is clearly presented, and it describes in detail, an approach which will be of significant interest to the readers of the journal. There are however a number of issues which would require to be addressed before publication.

Figure 1A. Although these data may well be of some interest to scientists working in this specific area, it is simply reproducing the data previously described in Ying et al, and subsequent publications on this topic with unmodified wild-type cells. As a consequence this data should be replaced with the data relating to the cell lines generated for this study or removed from the manuscript.

We have swapped Figure 1A for Supporting Information Figure 1. Figures 1A and 1B now show the FACS profiles of the wild type ESCs generated in this study. We decided to include the FACS profiles in the manuscript as we believe that the 2i medium provides better quality ESCs as compared to classic culture conditions based on the expression of the three core ESC transcription factors, in particular Nanog. We believe that the success of the GEMM-ESCs approach is largely attributed to the 2i medium; therefore, highlighting the importance of these culture conditions seems relevant.

Figure 1B-E, the differences between morula and blastocyst injection and already well established. This data is again generated from wild-type cells and I don't see the point of reproducing it here, especially when the majority of the animals discussed in this study (including those generated from all of the targeted ES cell lines) were generated by blastocyst injection.

We aim to present the GEMM-ESC approach as a method that can be applied to all mutant mouse strains, irrespective of the strain background. As re-deriving ESCs from various strain backgrounds may require optimizations at the micro-injection side, we find it relevant to show our optimization for wild type ESC clones from two different mouse inbred strains. While the chosen approach for optimization (i.e. morula vs. blastocyst injection) is not novel, our example accentuates the need for this type of optimization it in the GEMM-ESC approach.

Most of the micro-injections shown in this study were performed by blastocyst injection but not all as mentioned by the referee; see the five *Col1a1-frt* targeted C57BL/6J ESC clones injected in FVB/n morulae. Results are shown in Supporting Information Figure 5B and Supporting Table 1.

Figure 2 C and D are unnecessarily complicated and detract from the message behind the data. The comparison of coat colour to genetic contribution in each tissue simply informs how efficient the authors are at estimating contribution by coat colour. This data could simply be replaced by a more simple analysis which estimates the average contribution in chimeras 70% and above in each tissue or something similar.

Figure 2C and D have been simplified based on the referees advice. The figure legend is adjusted accordingly.

Figure 2 E and F and supporting information 2. It is quite clear there is a significant difference in the survival curve in the Kras-LSLG12D chimeras. It is not clear why this is in the supplementary data rather than the actual manuscript. The most likely explanation for this is that the ES cells have undergone a genetic change during the culture process, which is causing this reduction in survival. This should be discussed by the authors, as it is obviously highly relevant to the efficiency of this approach.

ESCs derived from Kras-LSLG12D mice only have a single mutant allele, therefore applying the GEMM-ESC approach to this genotype does not provide much time benefit as a single cross would be sufficient to introduce a new transgene. We included it, because this model is widely used and because it is backcrossed to a pure C57BL/6J background. Re-deriving ESCs from C57BL/6J and targeting them with the *Col1a1-frt* construct shows that the GEMM-ESC approach can also be applied to this commonly used mouse strain. However as we did not introduce a transgene in the Kras-LSLG12D background and for the limited benefit in time, we felt it was more suitable for the supporting information.

We do agree the shorter survival curve might be explained by a genetic change during the culture process. We have therefore added a sentence to the results section on page 8: "Alternatively, as the NSCLC chimeric cohort was produced from a single ESC clone, an unidentified genetic lesion might have been acquired during the re-derivation process that leads to accelerated tumour growth."

The authors analyse copy number variation in the targeted cell lines, but do not do so in the originally derived ES cell lines. It would be useful for them to do so as it may be that CNVs are observed equally frequently during the process of ES cell derivation. This may also explain the difference in the survival curve seen in the Kras chimeras.

We did perform copy number analysis on all originally derived ESC lines. In fact, all targeted ESC clones were analysed against the profile of the parental ESC line. We did not observe clear copy number variations (CNVs) in the originally derived ESC lines, although CNVs might have arisen during propagation and therefore exist as a minor subclone. An individual CNV (typically 1 copy gain) arising in a subpopulation of the originally derived ESC line will not be detectable by aCGH as it will disappear in the background. CNVs can only be identified once new subclones have been made, either by limiting dilution or by picking of clones after electroporation or transfection. In only one instance, we observed the same CNVs in two subclones of a parental line (CNV-3.1; CNV10.2), indicating these CNVs arose during ESC derivation. This was an exception, as the majority of targeted or subcloned ESCs had no CNVs or a unique CNV indicating that these alterations do not occur frequently during the process of ESC derivation.

Figure 3B should include the actual number of cell lines which are normal and those with CNVs, as it is hard to work out the actual figures from this graphical representation.

Figure 3B was adjusted and the actual number of normal cell lines is indicated on the side of each bar, whereas the total number of lines analysed is indicated below each bar. Due to lack of space, we could not indicate the actual numbers for the cell lines with 1, 2 or 3 CNVs, Trisomy or Lost Y. Precise numbers for these cell lines are listed in supporting information Table 3 and 4.

In figure 3C, 2 of the 3 cell lines analysed contain CNVs. The 3rd 'normal' cell line generated no chimeras from 88 injected blastocysts, suggesting this cell line is also carrying an undetected genetic alteration. Consequently in this analysis, these data imply that the vast majority of targeted cell lines (100%) are carrying genetic alterations. This is completely at variance with the way the data is described in the rest of the manuscript (e.g. figure 3b) and is obviously highly relevant to the efficiency with which this approach can be used. Again this should be highlighted and discussed by the authors.

We understand the concern. However, CNVs are also present in normal ESC culture and targetings, and are not specific to our approach (Liang et al., PNAS 2008 105:17453-6). We have included a comment in the discussion to highlight that this issue exists and could have an effect:

"Thus far, we did not observe any phenotypic changes in our chimeras or their offspring that could be attributed to a particular CNV; however, this remains a possibility. Also, aCGH screening may not allow for full identification of all aberrations that occur during *in vitro* culture as some apparently normal clones failed to give rise to chimeras indicating an underlying genetic or epigenetic defect. It should be noted that this issue is not specific for the approach described here but plays a role in any experiment using cloned ES cells (Liang et al., PNAS 2008 105:17453-6)".

The data generated in figure 4C comparing the survival curve of chimeras expressing the MycL1 cDNA with that of controls, contracts with the data derived from the F1 animals. Again this represents a big potential limitation of the system the authors describe as it clearly shows that significant differences generated in genetically modified animals cannot be replicated in chimeras. This issue should be addressed by the authors.

The MycL1 survival curve for the F1's is indeed sharper as compared to the chimeras, although we doubt this represents a significant difference. Still we see the point of referee #3. We have therefore provided a more nuanced discussion on the use of chimeras on page 15 of the revised manuscript:

"We show that the tumour characteristics of chimeric mice are very similar to those of conventional mice (Fig 2), though the tumour latency can differ depending on the model and level of chimerism (Fig 4C, Supporting Information Fig 2). Typically, the level of chimerism is estimated on basis of coat-colour contribution, although this consistently results in an overestimation of the true chimerism in the various tissues. In our experience 70-100% chimeras give a quite consistent reproducible tumour phenotype when the penetrance is high. In GEMMs with low penetrant phenotypes it is advisable to backcross the chimeras to the parental strain and use the F1 cohort. We feel that a more quantitative analysis on a particular tissue, e.g. tail, does not provide a substantial advantage above estimating chimerism on the basis of coat colour, as variations can also be found among different tissues."

In figure 4F, although apparently not statistically significant, there is a difference in percentage terms between the Rb;Trp53 mice and the Rb;Trp53;inv-Cag-Luc mice which are supposed to be a negative control. The reason behind this large difference should be addressed.

Although there seems to be a trend towards more MycL1 copy number gains in the $Rb1^{F/F}$;$Trp53^{F/F}$ group versus the $Rb1^{F/F}$;$Trp53^{F/F}$;invCag-Luc group, this difference is not statistically significant. We therefore feel that it is inappropriate to speculate on a potential difference between these two control groups. We prefer to restrict our discussion to the key point of this figure panel, i.e. MycL1 amplifications are hardly observed in MycL1 overexpressing tumours.

It appears that there is a consistent reduction of the percentage chimerism obtained at each stage of the process (compare figure 2A and B with figure 3A), although this does not appear to effect germline transmission. Again this may have a big effect on the utility of this approach and should be discussed by the authors. It also implies that there is a limit to the number of modifications which could be made to each.

The steady reduction in quality of chimeras after genetic engineering is to be anticipated and is not specific to our approach. Consecutive targetings in conventional ESC lines suffer from similar issues. In the legend of Fig 5, we have included a practical guideline that should overcome the issues raised here:

"In practice, we advise that for each model (i) multiple *Col1a1-frt* targeted GEMM-ESC clones are screened for their ability to efficiently generate high quality chimeras, (ii) two of the best-performing clones are selected for the Flp-in procedure, and (iii) at least two transgene-coding GEMM-ESC clones are used to generate cohorts. The final clones should originate from different *Col1a1-frt* targeted parental clones to minimize the chance of miss-interpreting phenotypes due to possible unwanted genetic alterations introduced by long-term culture. The selection of best-performing *Col1a1-frt* targeted GEMM-ESC clones is crucial for the efficiency to later generate experimental cohorts as the number of chimeras born per injected embryo is likely to decline after additional manipulations and propagation in culture."

The authors suggest that this approach would be frequently used to generate F1 cohorts of mice. The author's should probably point out that this would considerably reduce the range of applications of this technology, as it does not allow homozygous KO animals to be analysed.

On page 15 of the manuscript we state: "Indeed, a single cross of the chimeras to the original strain will result in F1 mice with or without the introduced construct". This means that by crossing the mice to the original strain background homozygosity of the KO alleles will be maintained while the modified allele will be either present (heterozygous) or absent. We have no intention of crossing our chimeras to wild type mice.

Also they make reference to using this system for genetic screen. The observed number of CNVs would suggest that the false positive rate would be extremely high using such an approach. This should be discussed in the text.

As in every experiment controls are crucial. The utility of the GEMM-ESC approach for genetic screens is shown in figure 4C. A comparison has to be made between chimeras with the *invCag-Luc* construct and the chimeras with the *invCag-geneX-Luc* construct. The difference in behaviour between these two lines will determine whether gene X modulates tumour behaviour. A CNV present in the parental *Col1a1-frt* GEMM-ESC clone will be shared and therefore cancelled out, but a CNV introduced at the time of Flp-in integration will be unique. To exclude potential effects of CNVs on tumour behaviour, it is advisable to perform the genetic screen in duplicate, using two independent ESC lines derived from independent embryos. To stress this point, we have included the following statement in the discussion: "It is, however, advisable to use two independent ESC lines as also recombinase-mediated introduction of constructs into the ESCs can give rise to chromosomal changes that could influence the outcome."

Minor points. I don't really see how this can be described as a pipeline, so reference to this should be removed and replaced by a more suitable word such a methodology or approach.

We have changed "pipeline" to "approach" throughout the manuscript.

In the panels relating to FACS analysis there are cell populations in blue and red. I am assuming the red population represent the isotype control but this should be clearly stated in the manuscript.

The Figure legend of 1A and B have been adjusted and now clearly state that the "Red population represents the isotype control".

Referee #4 (Remarks):

Genetically engineered mouse models (GEMMs) can provide important insights into the molecular basis of tumour development and enable testing of new intervention strategies, but their inherent complexity, combined with the time and expense it takes to development these models, has hampered their use as preclinical models for validating cancer genes and drug targets. In an attempt to accelerate target gene validation in mouse models the authors have developed an alternative strategy, which involves re-deriving embryonic stem cells (ESCs) from well-established and validated GEMMs. These GEMM-ESCs are then used for further genetic engineering either by classic gene targeting, gene editing or recombinase-mediated transgene integration. These GEMM-ESCs contain the same genetic modifications present in the original model plus the newly introduced genetic modification, such as the conditional overexpression of an oncogene or the conditional loss-of-expression of a tumour suppressor gene. The modified GEMM-ESCs can then be used to generate high quality chimeras that are likely to be equally susceptible to tumour induction as the original GEMM. The main advantage of this approach is its speed and flexibility, which makes it possible to comparatively analyse the phenotypic consequences of different genes and allelic series in a particular GEMM. Instead of crossing the chimeric mice to the desired strain and genetic background, ready-to-use GEMMS can now be produced on demand, which reduces the cost and total number of mice needed per experiment. This approach also allows for the establishment of a GEMM-ESC bank for the distribution ESCs with complex genotypes.

The feasibility of the GEMM-ESC production pipeline depends on reliable procedures and robust quality controls. In the experiments described here the authors have developed and validated such a pipeline for three GEMMs: two lung cancer models and one mesothelioma model. First, they developed and validated an efficient method for deriving ESCs from established GEMMs. Next, they introduced a Flp-in module just after the 3' UTR of the Col1a1 locus. This module serves as a docking sites, which permits the introduction of oncogenes or shRNAs for down-regulating tumour suppressor genes by Flp recombinase-mediated integration. This makes it possible to rapidly re-engineer the GEMM-ESCs by Flp-mediated recombination. Finally, they used one of their GEMM-ESC models to validate Mycl1 as a bona fide oncogene in small cell lung cancer.

Overall, this is a well done and through study, which provides compelling evidence supporting the use of GEMM-ESCs for rapid and high-throughput candidate cancer gene assessment and

validation. Over the past few years literally hundreds of new candidate cancer genes have been identified by cancer genome re-sequencing or through the use of mouse models. However, due to the shear number of genes being identified, classical methods for target validation, which include conventional transgenic and mouse knockouts, are not realistic. Faster and less costly methods like the one described here need to be developed. The one drawback of the method described here is the time it takes and the specialized expertise needed to make new GEMM-ESC models. Fortunately, these models can be easily distributed once they are made through distribution centres, such as the European Mouse Mutant Archive, once experts in the field generate them. In this regard, it is important to note that all three GEMM-ESC models described here will be made freely available through the European Mouse Mutant Archive. These models will no doubt be of great value to people who wish to validate candidate cancer genes identified in lung and mesothelioma cancer re-sequencing studies. While this manuscript is largely suitable for publication in EMBO Molecular Medicine in its present form there are a few minor issues that that authors need to address before publication.

Review Comments:

1. The authors need to more fully describe the rationale for using the Flp-in module and why it was targeted to the 3' UTR of Col1a1. There are other regions in the mouse genome where they could have targeted this module, such as Rosa26. Why was this particular locus picked? Also, why did they choose to use this module and not another module that uses for example Cre or some other docking site such as a lambda attachment site?

We included the following sentence on page 9 of the revised manuscript: We choose this system as it successfully applied by others {Yilmaz, 2012 #279; Zhu, 2009 #278}, allows for transgene induction in multiple somatic cell types {Carey, 2010 #280} and is compatible with a vector system for doxycycline-regulated, fluorescence-linked shRNAs {Dow, 2012 #273; McJunkin, 2011 #275; Premsrirut, 2011 #235}.

These were the reasons behind our choice to select this particular system. We are aware other systems exist each with their own advantages and disadvantages. We do not make the claim the *Col1a1* Flp-in system is superior to alternative approaches.

2. The correct strain designation is C57BL/6J and not C57Bl6J.

We have corrected the strain designation throughout the manuscript and figures.

3. What is meant by Cdkn2a/*? What do the *'s mean? The authors should use the correct locus designation to describe this mutant gene.*

See our answer to the third remark of referee #1.

4. In the Discussion the authors state that an ESC coat-colour contribution of at least 35% is required for efficient tumour induction studies. On what basis do they make this statement? It seems like the contribution required will dependent on how much the introduced oncogene or shRNA reduces the tumour latency. Some oncogenes or tumour suppressor genes might have a large effect on tumour latency while other might have a weaker effect. The required coat-colour contribution might thus vary depending upon the gene being validated. The authors should comments on this in the revised paper.

The 35% cut-off is based on Fig 4B. However, we acknowledge that this is a small basis on which to extrapolate a cut-off that can be applied to all GEMMs. Therefore, we have removed the following two sentences that refer to the cut-off point of 35%.

© EMBO

(1st sentence, from the discussion on page 13) – "taking into account that an ESC coat-colour contribution of at least 35% is required for efficient tumour induction studies (Fig 4B)."

(2nd sentence, from the legend to Figure 4B) – "Based on this experiment, we suggest a cut-off point of 35% coat-colour chimerism should be applied for inclusion of chimeras in tumour cohorts."

In addition, as a response to the first comment of referee #1 we have added the following statement to the discussion: Typically, the level of chimerism is estimated on basis of coat-colour contribution, although this consistently results in an overestimation of the true chimerism in the various tissues (Fig 2C-D). In our experience 70-100% chimeras give a quite consistent reproducible tumour phenotype when the penetrance is high. In GEMMs with low penetrant phenotypes it is advisable to backcross the chimeras to the parental strain and use the F1 cohort. We feel that a more quantitative analysis on a particular tissue, e.g. tail, does not provide a substantial advantage above estimating chimerism on the basis of coat colour, as variations can also be found among different tissues.

This statement also addresses the concerns raised here by referee #4.

2nd Editorial Decision 14 October 2013

Thank you for the submission of your revised manuscript to EMBO Molecular Medicine. We have now received the enclosed report from the referee who was asked to re-assess it. As you will see this reviewer is now globally supportive and I am pleased to inform you that we will be able to accept your manuscript pending the following final amendments:

1) Please address the issues highlighted by referee 3 in writing within the main text.
2) Editorial requests:

- please provide an accession number for the aCGH microarray dataset

Data of gene expression or copy number variation experiments described in submitted manuscripts should be deposited in a MIAME-compliant format with one of the public databases. We would therefore ask you to submit your microarray data to the ArrayExpress or GEO databases.

- please detail the statistical analyses performed in a special chapter within the materials and methods section of the manuscript.

I look forward to seeing a revised form of your manuscript as soon as possible.

***** Reviewer's comments *****

Referee #3 (Remarks):

The revised manuscript by Huijbers et. al describes the generation and validation of a method for generating mouse cancer models directly from embryonic stem cells. Overall the manuscript is worthy of publication and the data is clearly presented, and it describes in detail, an approach which will be of significant interest to the readers of the journal. There remain some issues which would require to be addressed before publication.

1. The authors raise the point that CNVs are present in normal ESC culture and add the following sentence to the text:

"It should be noted that this issue is not specific for the approach described here but plays a role in any experiment using cloned ES cells (Liang et al., PNAS 2008 105:17453-6)".

This statement is misleading as CNVs would not really affect the majority of experiments done by a

conventional ESC approach, as these would be bred out of the cohort. Using the method described in this study, where the cohort is generated directly from the ESCs and consequently the whole cohort would carry the same CNVs, this type of variation is likely to have a much more significant effect on the outcome of the experiments. This statement should be altered to reflect the fact that CNVs may present more of a problem using the GEMM-ESC approach.

2.Also the authors don't really address the main point that the significant amount of genetic variation introduced using this method could affect the outcome of experiments. 100% of the cell lines described here carry detectable extra genetic alterations in addition to the intended.

3. The authors suggest that this approach would be frequently used to generate F1 cohorts of mice. The author's should probably point out that this would considerably reduce the range of applications of this technology as it does not allow homozygous KO animals to be analysed.

This comment was really raising the point that it would not be possible to make a further knockout in a GEMM-ESC cell line, of a gene which was not altered in the original cell line. Obviously in this case it would not be possible to cross it to a heterozygous animal as the authors suggest.

All of these points can be addressed through textual changes

2nd Revision - authors' response 22 October 2013

We have made the following amendments in the text in response to referee #3. Addition "A" addresses the issues raised in point 1 and 2, and addition "B" the remark made in point 3.

A) On page 14 we have added the following sentence: *, although in an conventional ESC approach these CNVs are likely bred out of the cohort which is not the case when chimeras are used directly, although use of independent ESC clones can further reduce this risk.*

B) On page 15 we have added the following sentence: *Moreover, in the case where full target gene silencing is required to observe a phenotype, the highly efficient gene editing tools, TALENS and CRISPR/Cas, can be applied on GEMM-ESCs to generate a homozygous null allele that can be directly evaluated in chimeras (Fig 5).*

Furthermore we addressed the editorial requests. The aCGH microarray dataset is uploaded to ArrayExpress, accession E-MEXP-3998, and referred to in the Materials and Methods under section "Array Comparative Genome Hybridization". You can review this dataset with Username: Reviewer_E-MEXP-3998 and Password: xzkdcojd. Also a section on the statistical analysis is added to the Materials and Methods.

I hope these changes are satisfactory.
