# Supplementary material for: Rapid target gene validation in complex cancer mouse models using re-derived embryonic stem cells
Source: EMBO Mol Med. 2014 Jan 15;6(2):212–25. doi: 10.1002/emmm.201303297 (PMC3927956; doi:10.1002/emmm.201303297)
Supplement: Supplementary file 2 [file emmm0006-0212-sd2.pdf]

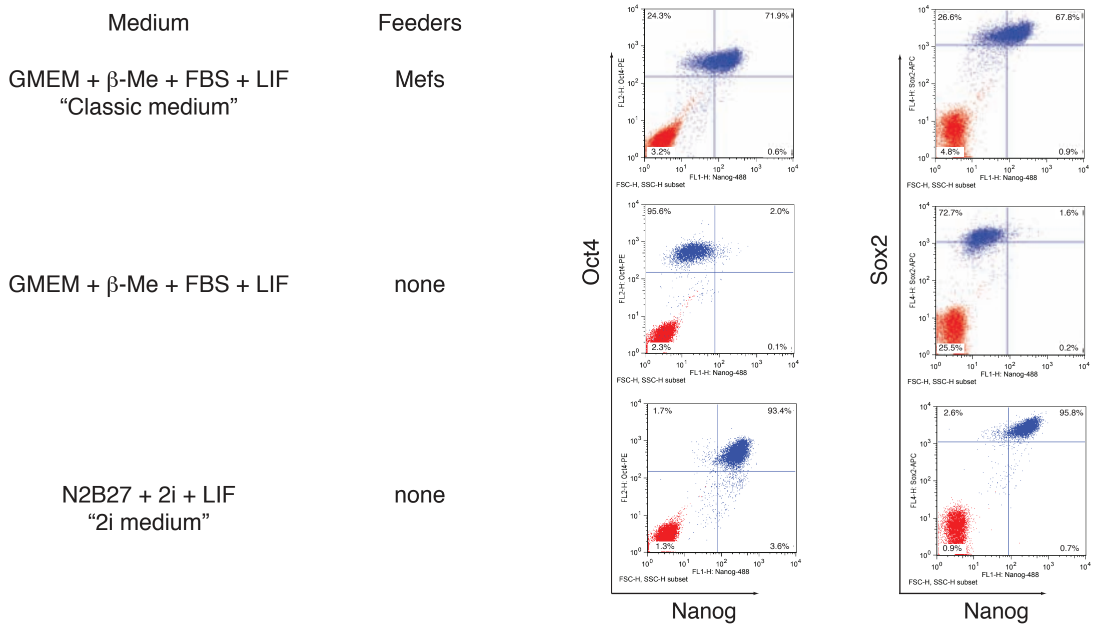

Supporting Information Figure 1. Expression of stem cell markers in ESCs cultured under different conditions. FACS analysis for three core ESC transcription factors, Nanog, Oct4 and Sox2 in IB10 ESCs (Robanus-Maandag et al, 1998) (129/Ola background) cultured in the classic and 2i medium. Almost all ESCs (93.4%) cultured in 2i medium highly expressed all three transcription factors and could be considered naïve ESCs capable of contributing to any germ layer. Nanog expression was absent in 24.3% of the ESCs cultured in the classic conditions and almost entirely lost upon removal of feeders.
