# Supplementary material for: Rapid target gene validation in complex cancer mouse models using re-derived embryonic stem cells
Source: EMBO Mol Med. 2014 Jan 15;6(2):212–25. doi: 10.1002/emmm.201303297 (PMC3927956; doi:10.1002/emmm.201303297)
Supplement: Supplementary file 3 [file emmm0006-0212-sd3.pdf]

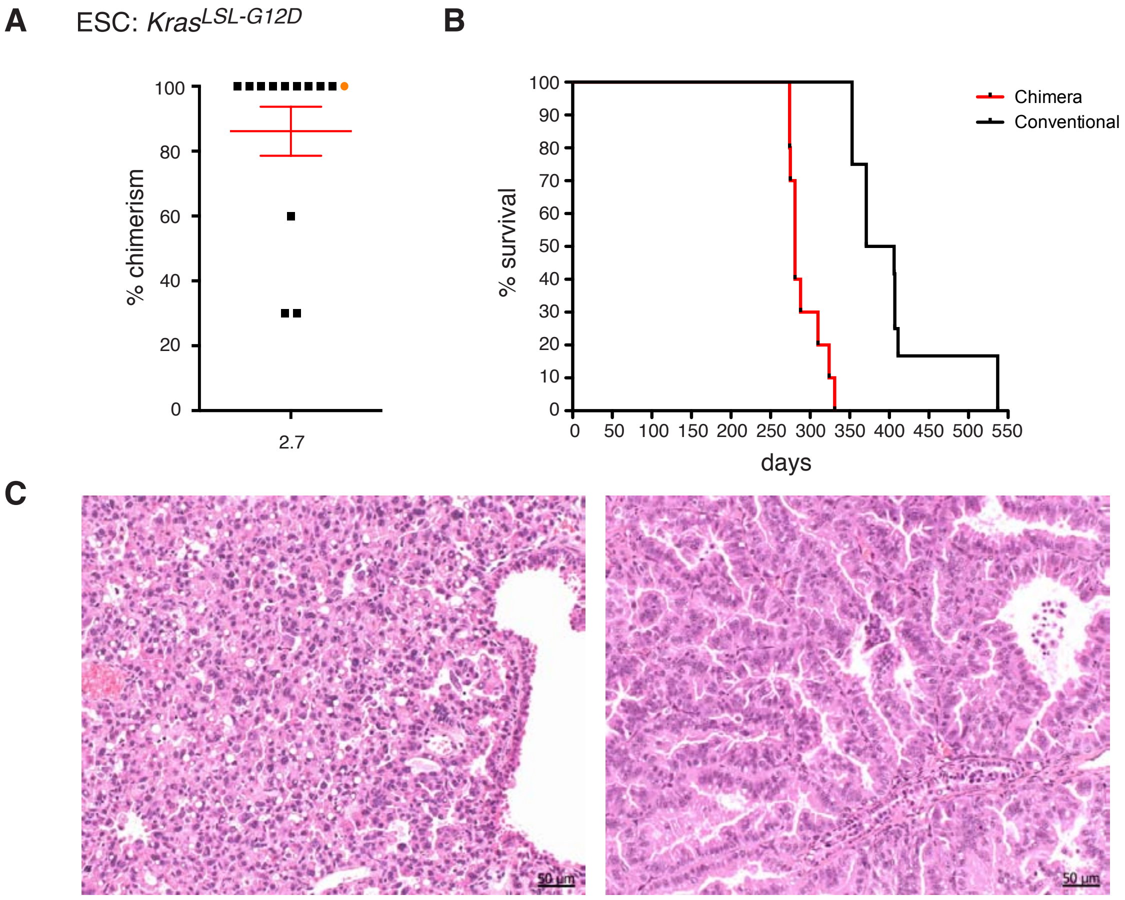

Supporting Information Figure 2. Validation of *Kras$^{LSL\text{-}G12D}$* ESCs and chimeras.

A. *Kras$^{LSL\text{-}G12D}$* ESC clone 2.7 was injected into FVB/n morulae and scored for its chimeric contribution. The majority of chimeras, 10 of 13, were entirely black (100% chimerism). ■ male, ● female.

B. Survival curves of *Kras$^{LSL\text{-}G12D}$* mice intratracheally injected with Ad5-Cre. The chimeras developed NSCLC with shorter latency as compared to the conventional *Kras$^{LSL\text{-}G12D}$* mice. Black line – conventional mice, Red line – chimeras.

C. Representative HE-stained sections of an adenocarcinoma (left panel) and a papillary carcinoma (right panel) in the lungs of chimeric mice.
