# Supplementary material for: Rapid target gene validation in complex cancer mouse models using re-derived embryonic stem cells
Source: EMBO Mol Med. 2014 Jan 15;6(2):212–25. doi: 10.1002/emmm.201303297 (PMC3927956; doi:10.1002/emmm.201303297)
Supplement: Supplementary file 4 [file emmm0006-0212-sd4.pdf]

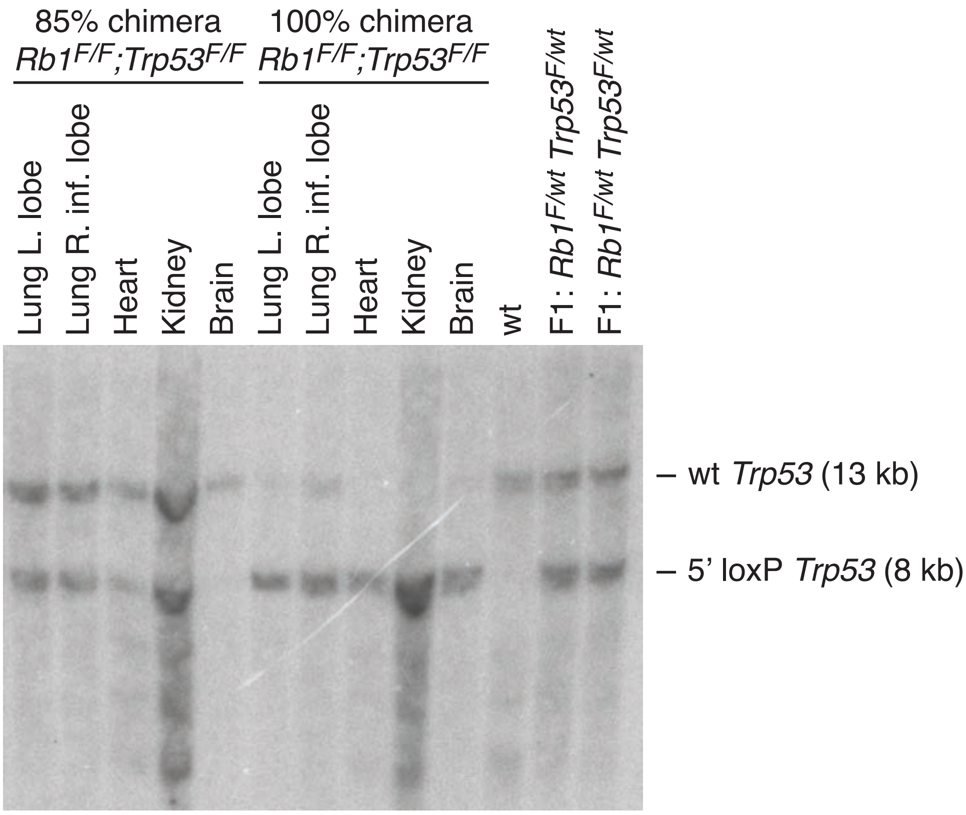

Supporting Information Figure 3. Genetic chimerism evaluated by Southern blot analysis. Example of a Southern blot performed on genomic DNA extracted from five organs of two *Rb1*$^{F/F}$;*Trp53*$^{F/F}$ chimeras to determine the level of genetic chimerism with a probe that distinguishes between a wildtype *Trp53* allele (13 Kb) or the floxed *Trp53* allele (8 Kb) reflecting the contribution by the host ESCs or cultured ESCs, respectively. Controls were genomic DNA extracted from a spleen of a wildtype mouse and genomic DNA of an F1 offspring of an *Rb1*$^{F/F}$;*Trp53*$^{F/F}$ chimera, therefore having one conditional *Trp53* allele and one wildtype *Trp53* allele.
