# Supplementary material for: Rapid target gene validation in complex cancer mouse models using re-derived embryonic stem cells
Source: EMBO Mol Med. 2014 Jan 15;6(2):212–25. doi: 10.1002/emmm.201303297 (PMC3927956; doi:10.1002/emmm.201303297)
Supplement: Supplementary file 5 [file emmm0006-0212-sd5.pdf]

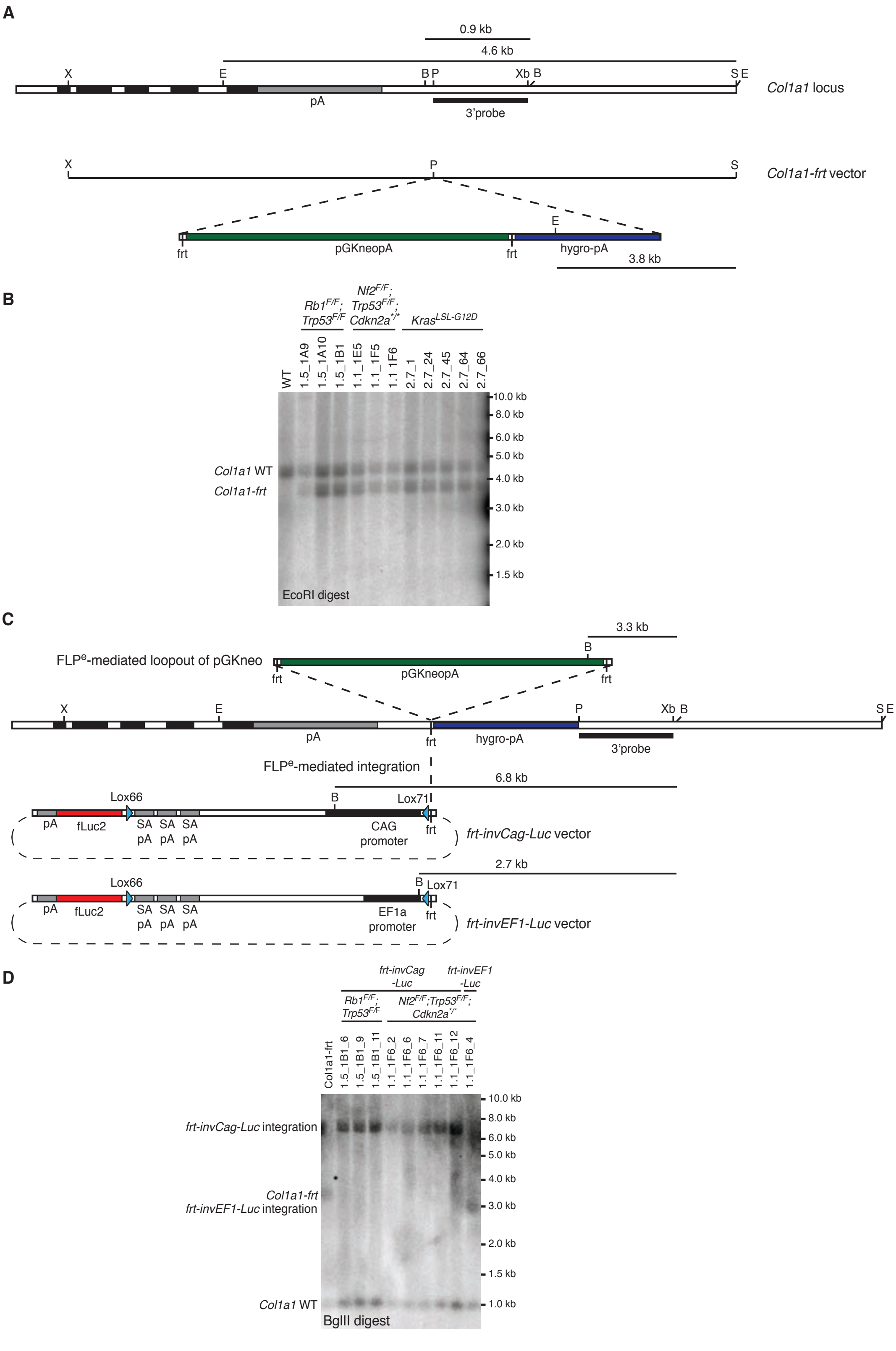

Supporting Information Figure 4. Genetic engineering in GEMM-ESC clones.

A. Introduction of *Col1a1-frt* construct in the *Col1a1* locus by homologues recombination as described by Beard et al., 2006. Schematic representation of the *Col1A1* locus and the *Col1a1-frt* targeting construct. The latter contains a Neomycin expression cassette flanked by frt sites, followed by an ATG-less Hygromycin resistance gene. The 3' internal probe is indicated. Exons are shown as black boxes and 3' UTR as a gray box. pA, polyadenylation signal. X, XhoI; E, EcoRI; B, BglII; P, PstI; Xb, XbaI; S, SpeI.

B. Southern blot analysis on *Col1a1-frt* targeted Neomycin-resistant ESC clones from three different genetic backgrounds using the 3' internal probe. Product sizes: *Col1a1-wt* - 4.6 kb and *Col1a1-frt* band - 3.8kb.

C. Introduction of the *frt-invCag-Luc* and the *frt-invEF1-Luc* vectors in *Col1a1-frt* targeted ESC clones using Flp recombinase, i.e. Flp-in strategy. Expression of Flp^{e} recombinase removes the pGK-neo-pA cassette from the targeted *Col1a1* locus and allows for Flp^{e}-mediated integration of vectors. ESC clones are selected for resistance to Hygromycin that is expressed by the ubiquitous promoters present in the *frt-invCag-Luc* and the *frt-invEF1-Luc* vectors. Both vectors contain Lox66 and Lox71 recombination sites that allow for promoter inversion after Cre recombinase expression causing firefly Luciferase expression. For clarity vectors are drawn linear and in the orientation they integrate in the genome. Note that the bacterial backbone, located between the splice acceptor/polyadenylation sites (SA pA) and the promoter, is integrated in the *Col1a1* locus after Flp-in reaction.

D. Southern blot analysis on Flp-in Hygromycin-resistant ESC clones from two different genetic backgrounds using a 3' internal probe. Product sizes: *Col1a1-wt* – 0.9 kb; *Col1a1-frt* band – 3.3 kb; *frt-invCag-Luc* integration band – 6.8 kb and *frt-invEF1-Luc* integration band – 2.7 kb.
