# Supplementary material for: Rapid target gene validation in complex cancer mouse models using re-derived embryonic stem cells
Source: EMBO Mol Med. 2014 Jan 15;6(2):212–25. doi: 10.1002/emmm.201303297 (PMC3927956; doi:10.1002/emmm.201303297)
Supplement: Supplementary file 6 [file emmm0006-0212-sd6.pdf]

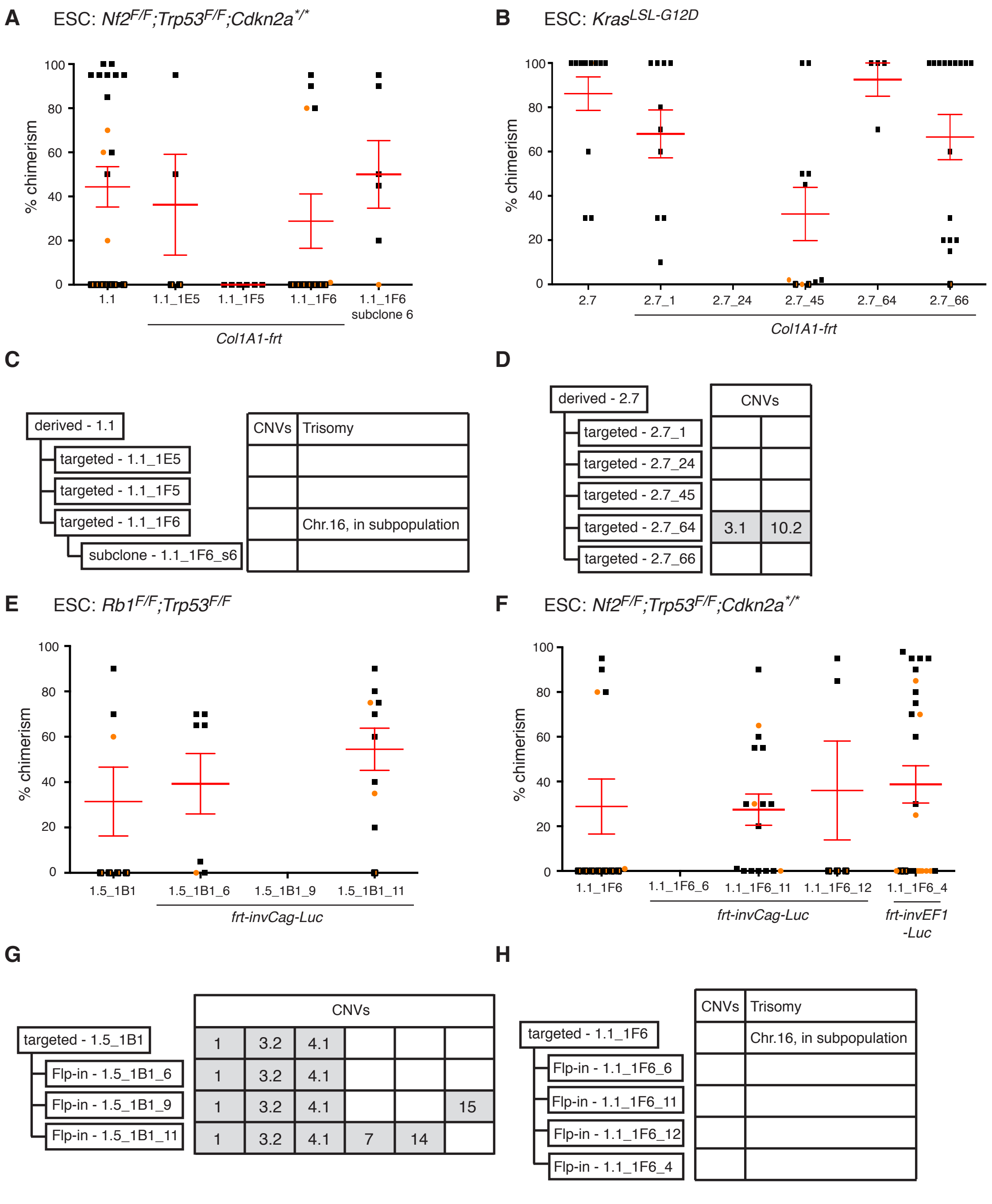

Supporting Information Figure 5. Genomic stability of GEMM-ESC clones after targeting and Flp-in.

A. Comparison of chimeric contribution between the parental *Nf2*$^{F/F}$*;Trp53*$^{F/F}$*;Cdkn2a*$^{*/*}$ ESC clone 1.1 and three *Col1a1-frt* targeted derivatives. Correct targeting was confirmed by Southern blot analysis using a 3' internal probe in the *Col1a1* locus (Supporting Information Fig 4B). Two *Col1a1-frt* targeted clones, i.e. 1.1_1E5 and 1.1_1F6, provided good and germline-competent chimeras (Supporting Information Table 1). Subcloning ESC clone 1.1_1F6 did not affect the ability to generate chimeric animals as shown by subclone 6. ■ male, ● female, ◧ n.d.

B. Comparison of chimeric contribution between the parental *Kras*$^{LSL\text{-}G12D}$ ESC clone 2.7 and five *Col1a1-frt* targeted derivatives. Correct targeting was confirmed by Southern blot analysis using a 3' internal probe in the *Col1a1* locus (Supporting information Fig. 4B). All *Col1a1-frt* targeted clones provided good and germline-competent chimeras, except for ESC clone 2.7_24 (Supporting information Table 1). ■ male, ● female, ◧ n.d.

C. aCGH analysis revealed no CNVs in *Nf2*$^{F/F}$*;Trp53*$^{F/F}$*;Cdkn2a*$^{*/*}$ ESC clones and its derivatives, only trisomy of chromosome 16 in a subpopulation of Col1a1-frt targeted ESC clone 1.1_1F6. The latter could be resolved by subcloning.

D. aCGH analysis revealed no CNVs in four *Col1a1-frt* targeted *Kras*$^{LSL\text{-}G12D}$ ESC clones, only targeted clone 2.7_64 had two independent CNVs reflecting single copy gains. CNV-3.1 encoded 5 genes, CNV-10.2: 13 genes (Details in Supporting Information Table 3).

E. Comparison of chimeric contribution between the *Col1a1-frt* targeted *Rb1*$^{F/F}$*;Trp53*$^{F/F}$ ESC clone 1.5_1B1 and three *frt-invCag-Luc* Flp-in derivatives. Correct Flp-in was confirmed by Southern blot analysis using a 3' internal probe in the *Col1a1* locus (Supporting Information Fig 4D). Two *frt-invCag-Luc* Flp-in clones, i.e. 1.5_1B1_6 and 1.5_1B1_11, provided good and germline-competent chimeras (Supporting Information Table 1). ■ male, ● female, ◧ n.d.

F. Comparison of chimeric contribution between the *Col1a1-frt* targeted *Nf2*$^{F/F}$*;Trp53*$^{F/F}$*;Cdkn2a*$^{*/*}$ ESC clone 1.1_1F6 and three *frt-invCag-Luc* Flp-in derivatives and one *frt-invEF1-Luc* Flp-in derivative. Correct Flp-in was confirmed by Southern blot analysis using a 3' internal probe in the *Col1a1* locus (Supporting Information Fig 4D). Two *frt-invCag-Luc* Flp-in clones, i.e. 1.1_1F6_11 and 1.1_1F6_12, provided good and germline-competent chimeras. Same for *frt-invEF1-Luc* Flp-in clone 1.1_1F6_4 (Supporting Information Table 1). ■ male, ● female, ◧ n.d.

G. aCGH analysis revealed three independent CNVs in two *frt-invCag-Luc* Flp-in *Rb1*$^{F/F}$*;Trp53*$^{F/F}$ ESC clones again only reflecting single copy gains. CNV-7 encoded 3 genes, CNV-14: 21 genes and CNV-15: 10 genes (Details in Supporting Information Table 3).

H. aCGH analysis on the Luciferase reporter *Nf2F/F;Trp53*$^{F/F}$*;Cdkn2a*$^{*/*}$ ESC clones revealed no additional chromosomal abnormalities.
