# Supplementary material for: Rapid target gene validation in complex cancer mouse models using re-derived embryonic stem cells
Source: EMBO Mol Med. 2014 Jan 15;6(2):212–25. doi: 10.1002/emmm.201303297 (PMC3927956; doi:10.1002/emmm.201303297)
Supplement: Supplementary file 7 [file emmm0006-0212-sd7.pdf]

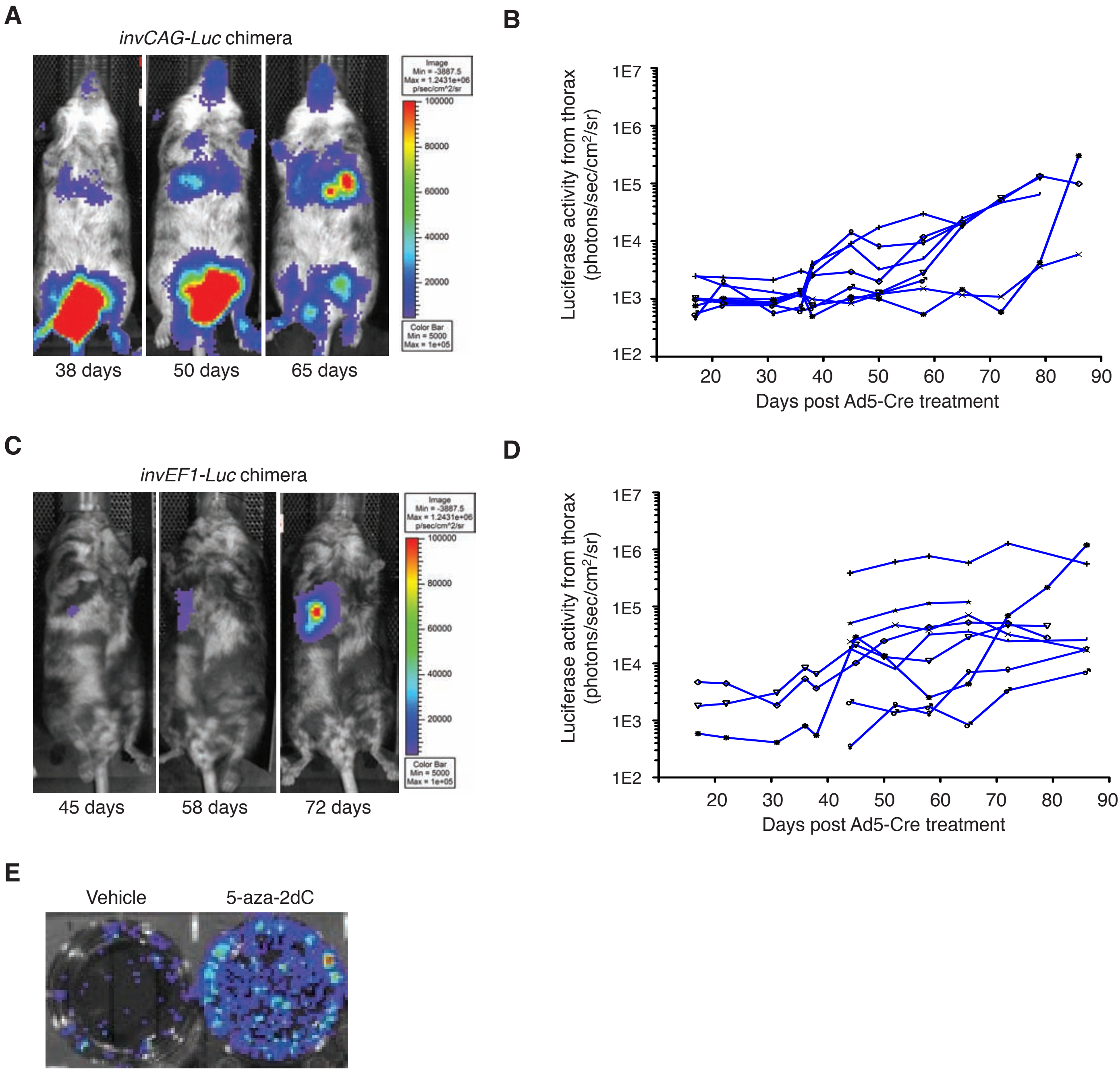

Supporting Information Figure 6. Luciferase imaging of mesothelioma in chimeras.

A. *In vivo* imaging of a *invCAG-Luc;Nf2*$^{F/F}$*;Trp53*$^{F/F}$*;Cdkn2a*$^{*/*}$ chimeric mouse injected intrathoracically with Ad5-Cre. Tumor growth was monitored weekly by imaging for Luciferase activity. Note, increased Luciferase signal emitted over time from the thorax reflecting mesothelioma growth *in vivo*, but also background signal from testis.

B. Luciferase activity emitted from the thorax of 8 chimeric *invCAG-Luc;Nf2*$^{F/F}$*;Trp53*$^{F/F}$*;Cdkn2a*$^{*/*}$ mice. Each line represents measurements of an individual mouse.

C. *In vivo* imaging of a *invEF1-Luc;Nf2*$^{F/F}$*;Trp53*$^{F/F}$*;Cdkn2a*$^{*/*}$ chimeric mouse injected intrathoracically with Ad5-Cre. Mesothelioma growth was monitored weekly by imaging for Luciferase activity. Note, the *frt-invEF1-Luc* construct showed little or no background signal in testis.

D. Luciferase activity emitted from the thorax of 9 chimeric *invEF1-Luc;Nf2*$^{F/F}$*;Trp53*$^{F/F}$*;Cdkn2a*$^{*/*}$ mice. Each line represents measurements of an individual mouse. Note, for the majority of mice, after an initial increase in Luciferase levels the signals leveled off up to the point the mice succumbed to malignant mesothelioma. It is likely that tumor growth was not accurately reflected by the *frt-invEF1-Luc* construct as the Luciferase signal was expected to increase steadily over time.

E. Transgene silencing by DNA methylation of EF1a promoter. A primary mesothelioma culture was extracted from a *invEF1-Luc;Nf2*$^{F/F}$*;Trp53*$^{F/F}$*;Cdkn2a*$^{*/*}$ chimeric mouse and treated for 2 days with demethylating agent 5-aza-2'-deoxycytidine (2 μM) at which point the cells reached confluency. This treatment led to an increase in Luciferase expression indicating the EF1a promoter was partially silenced by DNA methylation, possibly explaining the plateau in Luciferase levels observed *in vivo*.
