# Supplementary material for: Rapid target gene validation in complex cancer mouse models using re-derived embryonic stem cells
Source: EMBO Mol Med. 2014 Jan 15;6(2):212–25. doi: 10.1002/emmm.201303297 (PMC3927956; doi:10.1002/emmm.201303297)
Supplement: Supplementary file 8 [file emmm0006-0212-sd8.pdf]

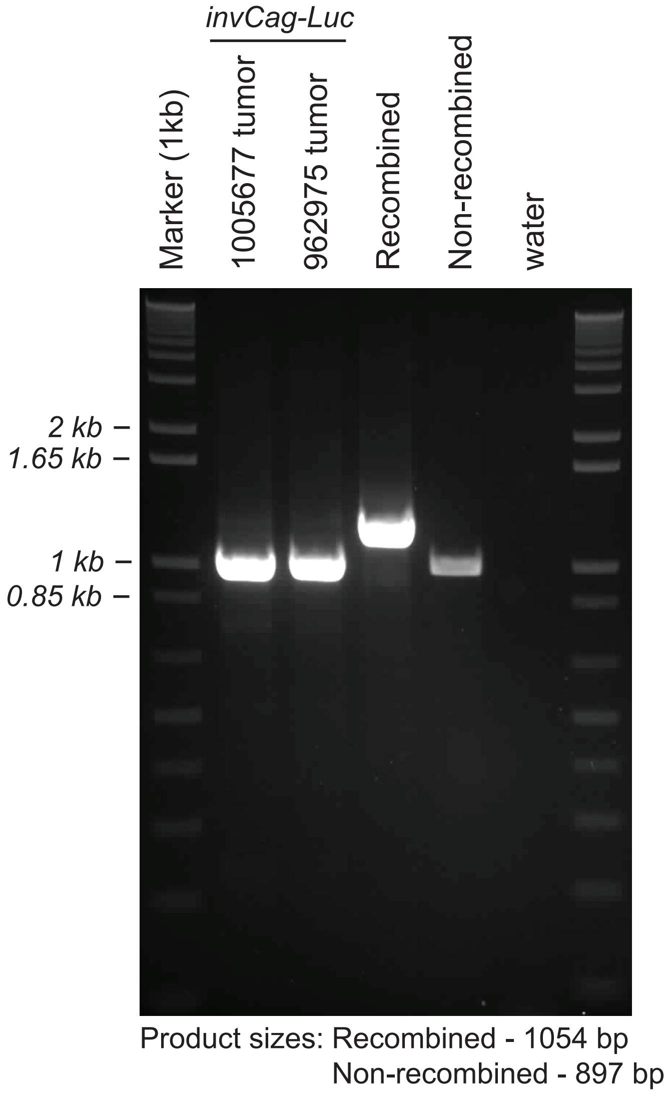

Supporting Information Figure 7: Absence of luciferase signal in two SCLC tumors is caused by failure to recombine the invCAG-Luc transgene.

Four *invCag-Luc;Rb1*$^{F/F}$*;Trp53*$^{F/F}$ mice, 1 out of 10 chimeras and 3 out of 12 F1's, developed SCLC without a Luciferase signal. Genomic DNA was extracted from two of these tumors (from chimera 962975 and F1 1005677), and a PCR was performed with three primers that distinguish the recombined *invCag-Luc* transgene from the non-recombined. Three primers were used: a forward primer on Lox66 `5′-GGCCGGCCATAACTTCGTATAATG-3′` and two antisense primers: one located in the vector backbone: `5′-CTGCGTTATCCCCTGATTCTGTGG-3`' to detect the non-recombined allele (product size: 897 bp) and one in the Hygromycin-B gene `5′-CCTACATCGAAGCTGAAAGCACGAG-3′` to identify the recombined allele (product size: 1054 bp). The PCR was performed with 1x Phusion Flash high-fidelity PCR mix (Thermo Scientific) at annealing and extension temperature of 72°C.
