# Supplementary material for: Rapid target gene validation in complex cancer mouse models using re-derived embryonic stem cells
Source: EMBO Mol Med. 2014 Jan 15;6(2):212–25. doi: 10.1002/emmm.201303297 (PMC3927956; doi:10.1002/emmm.201303297)
Supplement: Supplementary file 10 [file emmm0006-0212-sd10.pdf]

Supporting Information Table 1. GEMM-ESC generate chimeras after both targeting and Flp-in.

Genotype	Strain	ESC clone	Construct	MGI	No. of embryos injected[$]	No. born	No. of chimeras		GLT[#]
							M	F	
wt	C57BL/6J	1.3			46 B	22	7	10	0/4
		1.4			63 B	36	18	7	1/4
		1.4			159 M-	13	2	0	n.d.
		1.4			72 M	25	16	2	2/4
wt	FVB/n	1.2			23 B	10	5	3	2/5
		1.3			48 B	18	10	3	3/10
		1.3			38 M	6	4	0	2/3
Kras $^{LSL-G12D}$	C57BL/6J	2.7			106 M	13	12	1	2/3
Rb1$^{F/F}$;*Trp53*$^{F/F}$	FVB/n; 129/Ola	1.5			45 B	6	6	0	3/3
		1.10			39 B	10	6	3	2/2
		1.14			62 B	24	14	2	5/5
Nf2$^{F/F}$;*Trp53*$^{F/F}$;*Cdkn2a*$^{*/*}$	FVB/n; 129/Ola	1.1			49 B	23	10	3	4/4
		1.1			65 M	5	3	0	3/3
		1.5			82 B	22	11	0	2/2
Kras $^{LSL-G12D}$	C57BL/6J	2.7_1	*Col1A1-frt*	5443860	60 M	10	10	0	3/3
		2.7_24	*Col1A1-frt*	5443860	48 M	0	-	-	-
		2.7_45	*Col1A1-frt*	5443860	126 M	10	7	1	1/2
		2.7_64	*Col1A1-frt*	5443860	32 M	4	4	0	3/3
		2.7_66	*Col1A1-frt*	5443860	88 M	16	15	0	2/2
Rb1$^{F/F}$;*Trp53*$^{F/F}$	FVB/n; 129/Ola	1.5_1A9	*Col1A1-frt*	5443861	88 B	0	-	-	-
		1.5_1A10	*Col1A1-frt*	5443861	66 B	3	2	1	2/2
		1.5_1B1	*Col1A1-frt*	5443861	32 B	7	2	1	2/2
		1.5_1B1_r4	*Col1A1-frt*	5443861	62 B	15	8	1	2/2
Nf2$^{F/F}$;*Trp53*$^{F/F}$;*Cdkn2a*$^{*/*}$	FVB/n; 129/Ola	1.1_1E5	*Col1A1-frt*	5443866	29 B	4	2	0	n.d.
		1.1_1F5	*Col1A1-frt*	5443866	75 B	6	0	0	-
		1.1_1F6	*Col1A1-frt*	5443866	62 B	12	3	2	n.d.
		1.1_1F6_s6	*Col1A1-frt*	5443866	53 B	6	5	0	3/3
Rb1$^{F/F}$;*Trp53*$^{F/F}$	FVB/n; 129/Ola	1.5_1B1_6	*frt-invCag-Luc*	5443879	36 B	7	5	0	1/1
		1.5_1B1_9	*frt-invCag-Luc*	5443879	16 B	0	-	-	-
		1.5_1B1_11	*frt-invCag-Luc*	5443879	55 B	10	7	2	2/2
		1.5_1B1_r4_1	*frt-invCag-MycL1-Luc*	5499626	72 B	7	4	3	1/2
		1.5_1B1_r4_4	*frt-invCag-MycL1-Luc*	5499626	95 B	11	8	1	3/3
Nf2$^{F/F}$;*Trp53*$^{F/F}$;*Cdkn2a*$^{*/*}$	FVB/n; 129/Ola	1.1_1F6_6	*frt-invCag-Luc*	5443888	80 B	0	-	-	-
		1.1_1F6_11	*frt-invCag-Luc*	5443888	94 B	17	9	2	1/1[&]
		1.1_1F6_12	*frt-invCag-Luc*	5443888	89 B	5	2	0	1/1
		1.1_1F6_4	*frt-invEF1-Luc*	5443891	125 B	25	10	3	3/4

[$] B: ESCs injected into blastocysts. M-: ESCs injected into morulae without overnight culture. M: ESCs injected into morulae followed by overnight culture. [#] For wt genotypes, first litter was scored for transmission of coat color. For genetically modified genotypes, first litter was PCR screened for presence of mutant allele. [&] Germline transmission of mutant allele observed in second litter.
