# Supplementary material for: Rapid target gene validation in complex cancer mouse models using re-derived embryonic stem cells
Source: EMBO Mol Med. 2014 Jan 15;6(2):212–25. doi: 10.1002/emmm.201303297 (PMC3927956; doi:10.1002/emmm.201303297)
Supplement: Supplementary file 11 [file emmm0006-0212-sd11.pdf]

Supporting Information Table 2. Targeting and Flp-in efficiency

Genotype	Strain	Clone	Construct[#]	Positive clones of total	Recombination efficiency
wt	129/Ola	1B10	*Col1A1-frt*	13 of 36	36,1%
Kras $^{LSL\text{-}G12D}$	C57BL/6J	2.7	*Col1A1-frt*	36 of 96	37,5%
Rb1$^{F/F}$;*Trp53*$^{F/F}$	FVB/n;129/Ola	1.5	*Col1A1-frt*	11 of 32	34,4%
Nf2$^{F/F}$;*Trp53*$^{F/F}$;*Cdkn2a*$^{*/*}$	FVB/n;129/Ola	1.1	*Col1A1-frt*	54 of 156	34,6%
Rb1$^{F/F}$;*Trp53*$^{F/F}$;*Col1A1-frt*	FVB/n;129/Ola	1B1	*frt-invCag-Luc*	12 of 12	100%
Rb1$^{F/F}$;*Trp53*$^{F/F}$;*Col1A1-frt*	FVB/n;129/Ola	1B1_r4	*Frt-invCag-MycL1-Luc*	6 of 6	100%
Nf2$^{F/F}$;*Trp53*$^{F/F}$;*Cdkn2a*$^{*/*}$;*Col1A1-frt*	FVB/n;129/Ola	1F6	*frt-invCag-Luc*	12 of 12	100%
Nf2$^{F/F}$;*Trp53*$^{F/F}$;*Cdkn2a*$^{*/*}$;*Col1A1-frt*	FVB/n;129/Ola	1F6	*frt-invEF1-Luc*	4 of 4	100%

[#] Homology arms in targeting constructs were syngeneic to target loci in all GEMM-ESC clones.
