# Supplementary material for: Rapid target gene validation in complex cancer mouse models using re-derived embryonic stem cells
Source: EMBO Mol Med. 2014 Jan 15;6(2):212–25. doi: 10.1002/emmm.201303297 (PMC3927956; doi:10.1002/emmm.201303297)
Supplement: Supplementary file 12 [file emmm0006-0212-sd12.pdf]

Supporting Information Table 3. Description of CNVs

ID	GEMM-ESC			chr	Coordinates (Ensembl m37)		Length (Mb)	Number of genes	Genes Involved*
	Kras$^{LSL-G12D}$	*Rb1*$^{F/F}$;*Trp53*$^{F/F}$	*Nf2*$^{F/F}$;*Trp53*$^{F/F}$;*Cdkn2a*$^{*/*}$						
**CNV-1**		■		1	136193359	136645994	0,453	10	*Ppfia4; Tmem183a; Cyb5r1; Adipor1; Klhl12; Rabif; Kdm5b; Syt2*
**CNV-2**	■			2	92755257	93662725	0,907	13	*Syt13; Prdm11; Trp53i11; Tspan18; Cd82; Alx4; Ext2*
**CNV-3.1**	■			3	101890432	102249055	0,359	5	*Casq2; Vangl1*
**CNV-3.2**		■		3	96111178	96350050	0,239	7	*Rnu1b2; Terc; Hfe2*
**CNV-4.1**		■		4	123026875	123614240	0,587	18	*Macf1; Ndufs5; Akirin1; Rhbdl2; Mycbp; Rragc; Rragd*
**CNV-4.2**			■	4	129193347	129586376	0,393	18	*Hdac1; Lck; Fam167b; Eif3i; Dcdc2b; Iqcc; Ccdc28b; Txlna; Kpna6; Tmem39b; Khdrbs1; Ptp4a2*
**CNV-7**		■		7	103359146	103880790	0,522	3	*Odz4; Mir708*
**CNV-8**			■	8	86912339	87956740	1,044	46	*Cacna1a; Ier2; Nacc1; Trmt1, Lyl1; Nfix; SP1; Dand5; Gadd45gip1; Rad23a; Cair; Farsa; Syce2; Gcdh; Klf1; Dnase2a; Mast1; Rtbdn; Rnaseh2a; Prdx2; Junb; Hook2; Best2; Asna1; Tnpo2; Fbxw9; Dhps; Wdr83; Man2b1; Zfp791; Olfr371; Vps35; Orc6; Mylk3*
**CNV-10.1**			■	10	36853048	88392260	51,539	871	
**CNV-10.2**	■			10	41210244	41996548	0,786	13	*Ppil6; Cd164; Ccdc162; Cep57l1; Sesn1; Armc2; Foxo3*
**CNV-11**			■	11	61156901	62129943	0,973	18	*Slc47a1; Rnf112; Mfap4; Mapk7; B9d1; Epn2; Grap; Slc5a10; Fam83g; Prpsap2; Ulk2; Specc1; Adora2b; Zswim7; Ttc19*
**CNV-13**	■			13	54146552	54570201	0,424	5	*Drd1a; Sfxn1; Hrh2; Cplx2; Thoc3*
**CNV-14**		■		14	118536348	120906414	2,370	21	*Gpr180; Sox21; Abcc4; Cldn10; Dzip1; Dnajc3; Ugcgl2; Hs6st3; Oxgr1; Mbnl2; Rap2a*
**CNV-15**		■		15	84895118	85498105	0,603	10	*Smc1b; Ribc2; Fbin1; Atxn10; Wnt7b*
**CNV-16**		■		16	29396123	30388616	0,992	12	*Atp13a4; Opa1; Hes1; Cpn2; Lrrc15; Gp5*
**CNV-17**	■			17	44914119	46557867	1,644	39	Supt3h; Cdc5l; Spats1; Gm9104; Aars2; Tcte1; Tmem151b; Nfkbie; Slc35b2; Hsp90ab1; Slc29a1; Ent1; Capn11; Tmem63b; Mrpl14; Vegfa; Mrps18a; Rsph9; Mad2l1bp; Gtpbp2; Polh; Xpo5; Mir693; Polric; Yipf3; Tjap1; Dlk2; Abcc10; Zfp318
**CNV-X**	■			X	111588128	112022119	0,434	4	Klhl4; Ube2dnl

■ CNVs detected in GEMM-ESC clones

* Only some of the known genes are listed. The gene annotations are from Ensembl: www.ensembl.org/Mus_musculus/index.html)
