# Supplementary material for: Rapid target gene validation in complex cancer mouse models using re-derived embryonic stem cells
Source: EMBO Mol Med. 2014 Jan 15;6(2):212–25. doi: 10.1002/emmm.201303297 (PMC3927956; doi:10.1002/emmm.201303297)
Supplement: Supplementary file 13 [file emmm0006-0212-sd13.pdf]

Supporting Information Table 4. Distribution of CNVs in GEMM-ESC clones listed in Supporting Information Table 3

	GEMM-ESC clone	aCGH reference - ESC clone	Chimeras	Copy Number Variant: 1	2	3.1	3.2	4.1	4.2	7	8	10.1	10.2	11	13	14	15	16	17	X	lost Y	Trisomy
Kras$^{LSL-G12D}$	Col1A1-frt 1	2.7	Y																			
	Col1A1-frt 13	2.7																				
	Col1A1-frt 24	2.7	N																			
	Col1A1-frt 34	2.7																				
	Col1A1-frt 39	2.7													[blue]							
	Col1A1-frt 45	2.7	Y																			
	Col1A1-frt 64	2.7	Y			[pink]							[pink]									
	Col1A1-frt 66	2.7	Y																			
	Col1A1-frt 79	2.7			[blue]																	
	Col1A1-frt 90	2.7																				Chr.6 [pink]
	2.7 subclone 1	2.7																				Chr.15 [blue]
	2.7 subclone 2	2.7																				
	2.7 subclone 3	2.7																	[blue]			Chr.6 [red]
	2.7 subclone 4	2.7																				
	2.7 subclone 5	2.7																		[blue]		Chr.6 [pink]
	2.7 subclone 6	2.7																				Chr.6 [pink]
	2.7 subclone 7	2.7																				
	2.7 subclone 8	2.7																				Chr.6 [red]
	2.7 subclone 9	2.7																			[blue]	Chr.6 [red]
	2.7 subclone 11	2.7				[pink]							[pink]									
Rb1$^{F/F}$;*Trp53*$^{F/F}$	Col1A1-frt 1A9	1.5	N																			
	Col1A1-frt 1A10	1.5	Y															[blue]				
	Col1A1-frt 1B1	1.5	Y	[pink]			[pink]	[pink]														
	frt-invCag-Luc 6	1.5_1B1	Y	[pink]			[pink]	[pink]														
	frt-invCag-Luc 9	1.5_1B1	N	[pink]			[pink]	[pink]									[blue]					
	frt-invCag-Luc 11	1.5_1B1	Y	[pink]			[pink]	[pink]		[blue]						[blue]						
	Col1A1-frt 1B1 rederived 4	1.5_1B1	Y					[pink]														
Nf2$^{F/F}$;*Trp53*$^{F/F}$;*Cdkn2a*$^{F/F}$	Col1A1-frt 1E5	1.1	Y																			
	Col1A1-frt 1F5	1.1	N																			
	Col1A1-frt 1F6	1.1	Y																			Chr.16 [red]
	frt-invCag-Luc 1	1.1_1F6																				Chr.16 [pink]
	frt-invCag-Luc 2	1.1_1F6																			[blue]	Chr.16 [pink]
	frt-invCag-Luc 3	1.1_1F6							[blue]													
	frt-invCag-Luc 5	1.1_1F6																				Chr.16 [pink]
	frt-invCag-Luc 6	1.1_1F6	N																			
	frt-invCag-Luc 7	1.1_1F6																				Chr.16 [pink]
	frt-invCag-Luc 8	1.1_1F6									[blue]											
	frt-invCag-Luc 11	1.1_1F6	Y																			
	frt-invCag-Luc 12	1.1_1F6	Y																			
	frt-invEF1-Luc 1	1.1_1F6																				Chr.16 [pink]
	frt-invEF1-Luc 2	1.1_1F6																				
	frt-invEF1-Luc 3	1.1_1F6												[blue]								
	frt-invEF1-Luc 4	1.1_1F6	Y																			
	Col1a1-frt 1F6 subclone 1	1.1_1F6																				Chr.16 [pink]
	Col1a1-frt 1F6 subclone 3	1.1_1F6										[blue]										
	Col1a1-frt 1F6 subclone 5	1.1_1F6																				
	Col1a1-frt 1F6 subclone 6	1.1_1F6	Y																			

[blue] CNV detected once in GEMM-ESC clones

[pink] CNVs detected in multiple GEMM-ESC clones from the same parental clone

[red] Trisomy observed in a subpopulation of a GEMM-ESC clone
