# Supplementary material for: Rapid target gene validation in complex cancer mouse models using re-derived embryonic stem cells
Source: EMBO Mol Med. 2014 Jan 15;6(2):212–25. doi: 10.1002/emmm.201303297 (PMC3927956; doi:10.1002/emmm.201303297)
Supplement: Supplementary file 14 [file emmm0006-0212-sd14.pdf]

Supporting Information Table 5. Sequences of real time PCR primers

Primer name	Primer sequence
MycL1 F	5′- AGACTCAGGCCTGCTC -3′
MycL1 R	5′- GATTTCAAACAGCGGTAGATAG –3′
Actin F	5′- TTCAACACCCCAGCCATGTA -3′
Actin R	5′- TGTGGTACGACCAGAGGCATAC -3′
GapdH F	5′- TGGAAGATGGTGATGGGCTT -3′
GapdH R	5′- AACGACCCCTTCATTGACCT -3′
Tfrc F	5′- TGTAATTGGGTATAGGCTTGCA -3′
Tfrc R	5′- ATCCTGTACTCAAGAATTGGCT -3′
Tert F	5′- TCGAACAGCAAACCAACAGG -3′
Tert R	5′- AGAGACAGGCCCGAAGAAAA -3′
