# Supplementary material for: Rapid target gene validation in complex cancer mouse models using re-derived embryonic stem cells
Source: EMBO Mol Med. 2014 Jan 15;6(2):212–25. doi: 10.1002/emmm.201303297 (PMC3927956; doi:10.1002/emmm.201303297)
Supplement: Supplementary file 9 [file emmm0006-0212-sd9.pdf]

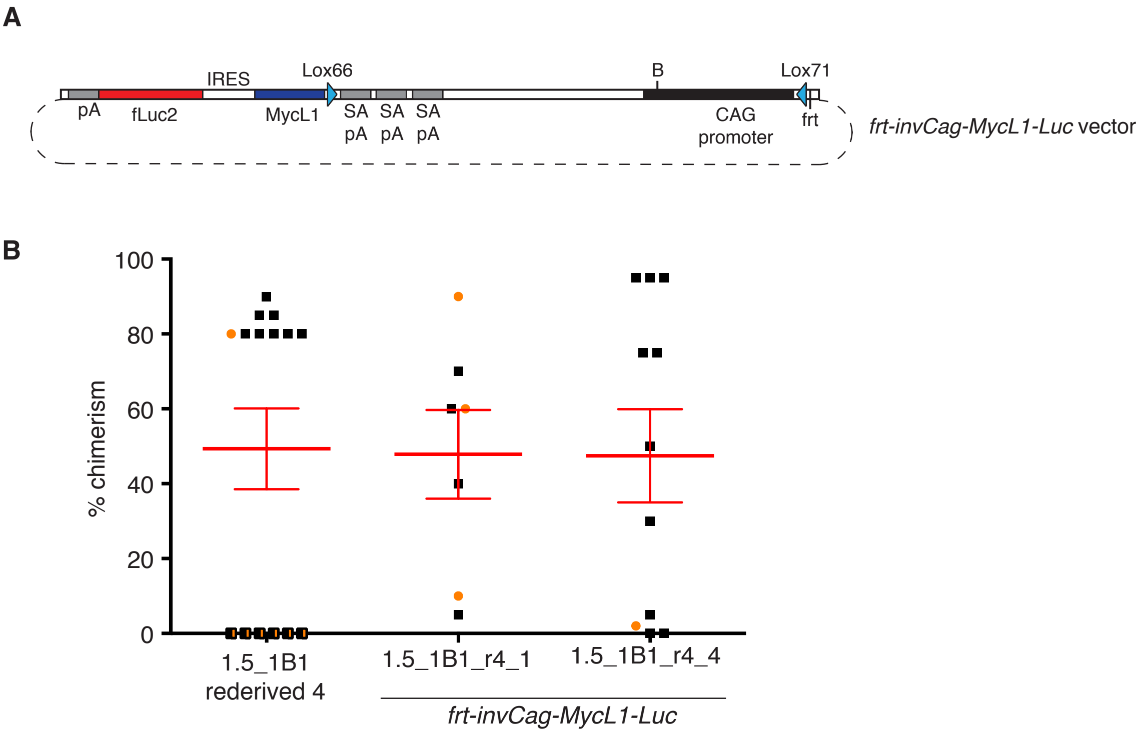

Supporting Information Figure 8: The MycL1 transgene and ESCs.

A. Schematic representation of the *frt-invCag-MycL1-Luc* vector. For clarity the vector is drawn linear and in the orientation it integrates in the genome. Note that the bacterial backbone, located between the splice acceptor/polyadenylation sites (SA pA) and the CAG promoter, is integrated in the *Col1a1* locus after Flp-in reaction.

B. Comparison of chimeric contribution between the parental $Rb1^{F/F}$;$Trp53^{F/F}$ ESC clone 1.5_1B1 re-derived 4 and two *frt-invCag-MycL1-Luc* Flp-in derivatives. Correct Flp-in was confirmed PCR screening. Both clones provided good and germline-competent chimeras (Supporting Information Table 1). ■ male, ● female, ◧ n.d.
